# Spatial transcriptomics reveals distinct role of monocytes/macrophages with high *FCGR3A* expression in kidney transplant rejections

**DOI:** 10.3389/fimmu.2025.1654741

**Published:** 2025-09-15

**Authors:** Yan Chen Wongworawat, Chirag Nepal, Mark Duhon, Wanqiu Chen, Minh-Tri Nguyen, Adam Godzik, Xinru Qiu, Wei Vivian Li, Gary Yu, Rafael Villicana, Craig Zuppan, Michael De Vera, Michael T. Eadon, Mark Haas, Charles Wang

**Affiliations:** ^1^ Department of Pathology and Human Anatomy, Loma Linda University Health, Loma Linda, CA, United States; ^2^ Center for Genomics, School of Medicine, Loma Linda University, Loma Linda, CA, United States; ^3^ Technology Center for Genomics & Bioinformatics, Pathology & Laboratory Medicine, University of California, Los Angeles, Los Angeles, CA, United States; ^4^ Transplant Institute, Loma Linda University Health, Loma Linda, CA, United States; ^5^ Division of Biomedical Sciences, University of California Riverside School of Medicine, Riverside, CA, United States; ^6^ Department of Statistics, University of California, Riverside, Riverside, CA, United States; ^7^ Mailman School of Public Health, Columbia University, New York, NY, United States; ^8^ Divisions of Nephrology and Clinical Pharmacology, Indiana University, Indianapolis, IN, Los Angeles, CA, United States; ^9^ Pathology and Laboratory Medicine, Cedars-Sinai Medical Center, Los Angeles, CA, United States

**Keywords:** spatial transcriptomic, kidney allograft antibody mediated rejection, cell mediated rejection, Fc gamma receptor IIIA (FCGR3A), monocytes, macrophages, innate immunity, trained immunity

## Abstract

**Introduction:**

Kidney transplant rejections are classified as active antibody mediated rejection (AMR) and cell mediated rejection (TCMR), with AMR primarily driven by antibodies produced by B cells, whereas TCMR is mediated by T lymphocytes that orchestrate cellular immune responses against the graft. Emerging evidence highlights the essential roles of innate immune cells in rejections, especially monocytes/macrophages and natural killer (NK) cells. However, the roles of specific innate immune cell subpopulations in kidney allograft rejection remain incompletely understood.

**Methods:**

We performed the spatial transcriptomics using the formalin-fixed paraffin-embedded (FFPE) core needle biopsies from human kidney allografts.

**Results:**

We demonstrated that non-rejection, AMR, acute TCMR and chronic active AMR have distinct transcriptomic features. Subclusters of monocytes/macrophages with high *Fc gamma receptor IIIA* (*FCGR3A*) expression were identified in C4d-positive active AMR and acute TCMR, and the spatial distribution of these cells corresponded to the characteristic histopathological features. Key markers related to monocyte/macrophage activation and innate alloantigen recognition were upregulated, along with metabolic pathways associated with trained immunity in AMR and TCMR.

**Discussion:**

Taking together, these findings revealed that intragraft monocytes/macrophages with high *FCGR3A* expression play a critical role in kidney transplant rejections.

## 1 Introduction

Allograft biopsy remains the gold standard for diagnosing kidney transplant rejections. International standard classification systems, Banff classification, define antibody-mediated rejection (AMR) and cell-mediated rejection (TCMR) in kidney transplants using specific histopathological and immunological criteria (1). AMR is classified into active, chronic active, and chronic forms. The diagnosis of AMR requires evidence of acute tissue injury - such as glomerulitis and peritubular capillaritis (collectively termed microvascular inflammation [MVI]), antibody interaction with the endothelium (C4d staining positivity), the presence of donor-specific antibodies (DSA), and chronic tissue injury (e.g. transplant glomerulopathy) (1). In contrast, TCMR is classified into acute and chronic active forms. The diagnosis and grading of TCMR are based on the degree of interstitial inflammation and tubulitis (1).

Mechanistically, AMR is primarily driven by antibodies produced by B cells, whereas TCMR is mediated by T lymphocytes that orchestrate cellular immune responses against the graft (2, 3). Increasing evidence highlights the essential roles of innate immune cells, especially monocytes/macrophages and natural killer (NK) cells, in solid organ transplantation (4–9, 10). Macrophages play pivotal roles in the innate immune response to transplant allografts during acute rejection by producing proinflammatory cytokines and generating reactive oxygen and nitrogen species (ROS and RNS) (6, 11). Both donor- and recipient-derived monocytes/macrophages activate adaptive immune responses by functioning as antigen-presenting cells (APC). They activate T cells through co-stimulatory signals, leading to release of pro-inflammatory cytokines and resulting in acute rejection (9). Macrophages are also implicated in chronic rejection and graft failure (9, 12, 13).

Reflecting these advances, the Banff classification is continually updated; for example, the Banff 2022 meeting introduced the entity of DSA-negative, C4d-negative, MVI, which may involve NK cell activation and other innate immune mechanisms (14). Additionally, the Banff system has incorporates molecular diagnostics, such as transcriptomic microarrays (e.g. Molecular Microscope [MMDX]) and Banff Human Organ Transplant Gene (B-HOT) panel) (15–17), to improve detection and classification of rejection beyond conventional histology. However, these techniques have limitations: the MMDX requires fresh frozen tissues, the B-HOT needs a high number of isolated cells – which can be challenging to obtain from clinical core needle biopsies - and both methods lack the ability to preserve spatial information (18). Spatial transcriptomics can overcome these limitations, by detecting RNA expression and mapping gene activity within a single hematoxylin and eosin-stained (H&E) - stained section from formalin-fixed paraffin-embedded (FFPE) tissue while preserving spatial context, revealing the distribution of various cell types and molecular pathways within their native microenvironments. This spatial information is particularly valuable in complex tissues like kidney allografts, where the location of immune cells relative to specific kidney structures can provide important diagnostic insights. Despite its promise, there is a paucity of research implementing spatial transcriptomics in transplantation studies (19–21). Furthermore, the spatial transcriptomic characteristics of monocytes/macrophages in kidney allograft rejection have not yet been fully investigated.

Leveraging the advantage of spatial transcriptomics, we performed spatial transcriptomic analysis on FFPE core needle biopsy samples from human kidney allografts representing various rejection groups to identify distinct monocytes/macrophages subclusters. Additionally, we conducted functional pathway and gene network analyses to elucidate the underlying biological, cellular, and molecular processes, with a particular focus on innate immune mechanisms.

## 2 Materials and methods

### 2.1 Human kidney allograft core needle biopsies case selection

We selected 8 cases based on histopathological and clinical features (**Table 1**), representing 4 diagnostic groups: 1) non-rejection conditions; 2) Active AMR; 3) Acute TCMR; 4) Chronic active AMR. The clinical diagnosis is interpreted by our renal pathologists based on the 2018 Banff Criteria (22).

**Table 1:** Histopathological and clinical features of cases.

Case #	1	2	3	4	5	6	7	8
Diagnostic category	Non-rejection		Active AMR		Acute TCMR		Chronic active AMR	
Pathologic diagnosis	Acute CNI toxicity	Subtle ATI	C4d-positive active AMR	C4d-negative active AMR	Acute TCMR, grade 1B, plasma cell rich	Acute TCMR, grade 2A	Chronic active AMR (Case #1)	Chronic active AMR (Case #2)
Banff Scores	t1, i0, v0, g0, ptc0, ci0, ct0, cg0, ti0, i-IFTA0, pvl0, C4d0	t0, i0, v0, g0, ptc0, ci0, ct0, cg0, ti0, i-IFTA0, pvl0, C4d0	t1, i1, v1, g2, ptc2, ci0, ct0, cg0, ti1, i-IFTA0, pvl0, C4d3	t0, i0, v0, g2, ptc2, ci0, ct0, cg0, ti0, i-IFTA0, pvl0, C4d1	t3, i3, v0, g0, ptc0, ci0, ct0, cg0, ti3, i-IFTA0, pvl0, C4d1	t3, i2, v1, g0, ptc2, ci0, ct0, cg0, ti2, i-IFTA0, pvl0, C4d1	t0, i0, v0, g2, ptc0, ci0, ct0, cg1b, ti0, i-IFTA0, pvl0, C4d2	t0, i0, v0, g1, ptc1, ci0, ct0, cg2, ti0, i-IFTA0, pvl0, C4d2
Age	41	58	53	31	28	36	41	49
Cause of ESKD	Hepato-renal syndrome	Diabetes	Unknown	Hypoplastic kidney	Unknown	Unknown	Unknown	Unknown
SCr (mg/dL)	1.5	1.9	6.1	1.5	18	10	1.36	1.4
DSA	Positive	Negative	Positive	Positive	Positive	Positive	Negative	Positive
Graft Function	DGF	DGF	Normal	Normal	Normal	Normal	Normal	Normal
Immuno-suppression Regimen	CSA, MMF, PRDL	TAC, MMF, PRDL	TAC, MMF, PRDL	TAC, MMF, PRDL	TAC, MMF, PRDL	TAC, MMF, PRDL	TAC, MMF, PRDL	TAC, MMF, PRDL

CNI, acute calcineurin inhibitors; AMR, active antibody mediated rejection; ATI, toxicity acute tubular injury; Banff Score: tubulitis (t), interstitial inflammation in non-scarred areas (i), intimal arteritis (v), glomerulitis (g), peritubular capillaritis (ptc), interstitial fibrosis (ci), tubular atrophy (ct), glomerular basement membrane double contours (cg), total inflammation (ti), inflammation in the area of IFTA (i-IFTA), polyomavirus load (pvl); CSA, Cyclosporine A; DGF, delayed graft function; DSA, donor specific antibody; ESKD, end-stage kidney disease; IFTA, interstitial fibrosis and tubular atrophy; MMF, mycophenolate mofetil; PRDL, prednisolone; Scr, Serum creatine; TAC, Tacrolimus; TCMR, cell mediated rejection.

### 2.2 Perform spatial transcriptomics using FFPE core needle biopsies of human kidney allografts

We performed 10x Genomic Visium spatial transcriptomics analysis on H&E - stained sections from archived FFPE core needle biopsies of human kidney allografts following Visium Spatial Gene Expression for FFPE workflow (Graphic Abstract). 1) Sample preparation and RNA quality control: section FFPE tissues onto charged glass slides. 2) Assess RNA integrity using methods Distribution Value 200 (DV200): DV200 represents the percentage of RNA fragments that are longer than 200 nucleotides in a sample. This method is particularly useful for evaluating the quality of degraded RNA samples, such as those extracted from FFPE tissue. Only samples with a DV200 value equal to or greater than 30% were processed. 3) Performed standard H&E staining directly on the glass slides. 4) Evaluated H&E staining slides to select areas of interest for 6.5 x 6.5 mm capture areas. 5) Probe Hybridization with whole transcriptome probe panels. 6) Used the Visium CytAssist instrument to precisely transfer bound probes onto the Visium slide. The Visium slide contains 6.5 x 6.5 mm capture areas with 55 μm barcoded squares. 7) Generated gene expression libraries from each tissue section (library preparation). 8) Sequenced the libraries on compatible Illumina sequencers, such as NovaSeq X series systems. 9) Employed Space Ranger software for data processing, applied standard quality control metrics to filter out low-quality spots, and combined all eight samples into a unified dataset (23–25). 10) Utilized Loupe Browser for interactive data exploration, integrating whole transcriptome analysis with precise spatial information from archived FFPE samples.

### 2.3 Differential gene expression, cluster identification and cell typing

All bioinformatics analysis was performed utilizing the BioTuring Lens platform (https://bioturing.com) (26, 27). 10X Visium spots were clustered via the Louvain method (principal component analysis (PCA) Resolution=1). Uniform Manifold Approximation and Projection (UMAP) visualization or t-distributed stochastic neighbor embedding (t-SNE) dimension reduction were generated via PCA of gene expression with no batch correction (n_neighbors=30). Segmentation analysis was applied to acquire 4–7 unsupervised clusters in each diagnostic category (**Supplementary Figure 1**). Cell types and subtypes per Louvain-derived cluster were predicted using the HaiTam algorithm (https://talk2data.bioturing.com). Spots that were not confidently characterized into a single cell type (i.e., undefined) were omitted from the analysis. UMAP-based visualization displayed clusters with annotated labels, which were obtained based on histopathologic features and known marker genes associated with kidney structures (**Supplementary Figure 1**) (28). Differential expression of genes (DEG) among spots in each case was calculated via the Venice algorithm (*p*<0.05) treating each spot as an individual sample data point. Hierarchical clustering heatmaps of the DEGs were generated and organized via a dendrogram of the cases and cluster plots of marker genes per cluster. Expression of specific genes per spot was measured and overlayed onto the UMAP or t-SNE.

### 2.4 Assessing concordance between FFPE tissue transcriptomic signatures and published RNA signatures of transplant rejection

To evaluate the consistency between our findings and existing research, we compared the transcriptomic signatures of AMR and TCMR from our FFPE tissue analysis with RNA signatures derived from frozen tissue bulk transcriptome microarrays, as reported by Halloran et al. in 2018 and 2024. This comparison was visualized using a Venn diagram, highlighting similarities and differences between the two approaches.

### 2.5 Functional pathway and gene network analysis

To analyze the gene networks, canonical, and bio-functional pathways, we applied Gene Ontology (GO) Enrichment Analysis tools to the lists of differentially expressed genes (ShinyGo v0.66, http://bioinformatics.sdstate.edu/go/) and Kyoto Encyclopedia of Genes and Genomes (KEGG) (29).

## 3 Results

### 3.1 Different rejection types displayed distinct transcriptomic signatures

**Figure 1:**
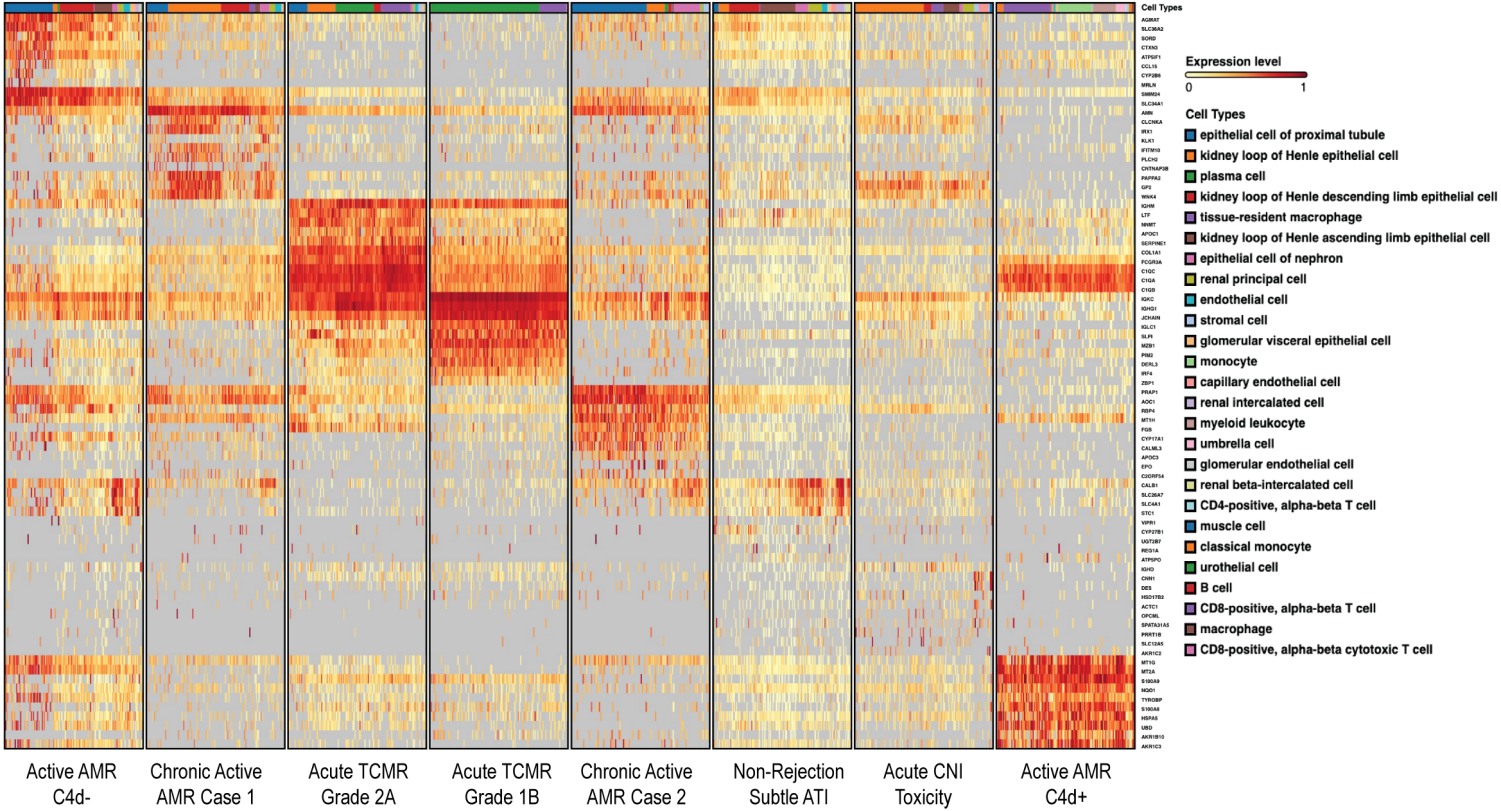
Different rejection types displayed distinct transcriptomic signatures. The heatmap, generated on the BioTuring platform, displayed differentially expressed genes (DEGs) organized via a dendrogram that illustrated the hierarchical relationships between cases. Color intensity represented gene expression levels, with red shades indicating higher expression and yellow shades indicating lower expression. The hierarchical clustering of rows (genes) and columns (cases) illustrated gene expression differences among four different diagnostic groups. These conditions exhibited distinct gene expression patterns, except for active AMR which demonstrated significantly different transcriptomic signatures between C4d-negative and C4d-positive active AMR cases. Chronic active AMR shared some overlapping features with acute TCMR. The dendrogram provided a visual representation of the genetic similarity and divergence among the studied cases.

To identify DEGs in each of the rejection types with respect to non-rejection conditions, we used the Venice algorithm. Hierarchical clustering of the DEGs revealed distinct gene expression pattens among 8 cases (**Figure 1**). Similar transcriptomic profiles patterns were observed among two cases in the same diagnostic groups (non-rejection cases, acute TCMR and chronic active AMR), except for the active AMR group. The C4d-positive active AMR case demonstrated significantly different transcriptomic signatures compared to the C4d-negative active AMR case, despite both being positive for donor-specific antibodies (DSA). Moreover, C4d-negative active AMR case showed a closer pattern to chronic active AMR cases. Furthermore, chronic active AMR cases shared some overlapping features with acute TCMR, which is consistent with recent study published by Shah et, al (30). These results demonstrated that the transcriptomic signatures from FFPE core needle biopsy tissues have the potential to aid in distinguishing between different types of rejection and may also enable further subclassification of AMR.

To evaluate the concordance between DEG derived from our FFPE tissue transcriptomic signatures with top transcripts associated with rejection by MMDX (16, 31), we compared two gene sets and observed some overlapping between our FFPE tissue transcriptomic signatures associated with active AMR and acute TCMR, and the MMDX transcripts linked to universal rejection (16) (**Supplementary Figures 2A–C**). In addition, our FFPE tissue transcriptomic signatures associated with active AMR and acute TCMR showed some overlapping with the top 20 transcripts linked to AMR, TCMR, and injury- and rejection-associated transcripts as reported by Halloran et al. in their 2024 MMDX study (**Supplementary Figures 2D–F**).

Furthermore, our analysis of the top 30 transcriptomic signatures in FFPE tissue from rejection groups (**Supplementary Table 1**) revealed additional important genes that are associated with transplant rejection. For example, in C4d-positive active AMR case, *S100A8* and *S100A9* were significantly upregulated. These calcium-binding proteins, primarily expressed in monocytes, play a crucial role in kidney transplant rejections, and high expression levels of S100A8 and S100A9 in myeloid cells during kidney transplant rejections have been linked to favorable outcomes (32). In acute TCMR, the expression of *FCGR3A* gene, which encodes the Fc gamma receptor IIIA (FcγR IIIA or CD16), was significantly increased, with its specific role to be elaborated upon later. Additionally, Interferon Regulatory Factor 4 (*IRF4*) was significantly upregulated. Similar to *IRF1*, this transcription factor is critical for immune regulation, particularly in T and B cells, and plays a significant role in transplant rejection by regulating genes involved in inflammation and lymphocyte activation (33). *IRF4* not only regulates adaptive immune responses but also plays a crucial role in the function and differentiation of innate immune cells such as monocytes and macrophages (34). For example, IRF4 negatively modulates proinflammatory cytokine production by macrophages following Toll-like receptor stimulation, underscoring its vital regulatory role in innate immunity (35). Moreover, the expression of complement component C3 was significantly increased. C3, part of the complement system that is frequently activated in acute AMR (33), was also significantly increased in acute TCMR.

### 3.2 Distinct subclusters of monocytes/macrophages exhibiting high FCGR3A expression were identified in acute rejection groups

**Figure 2:**
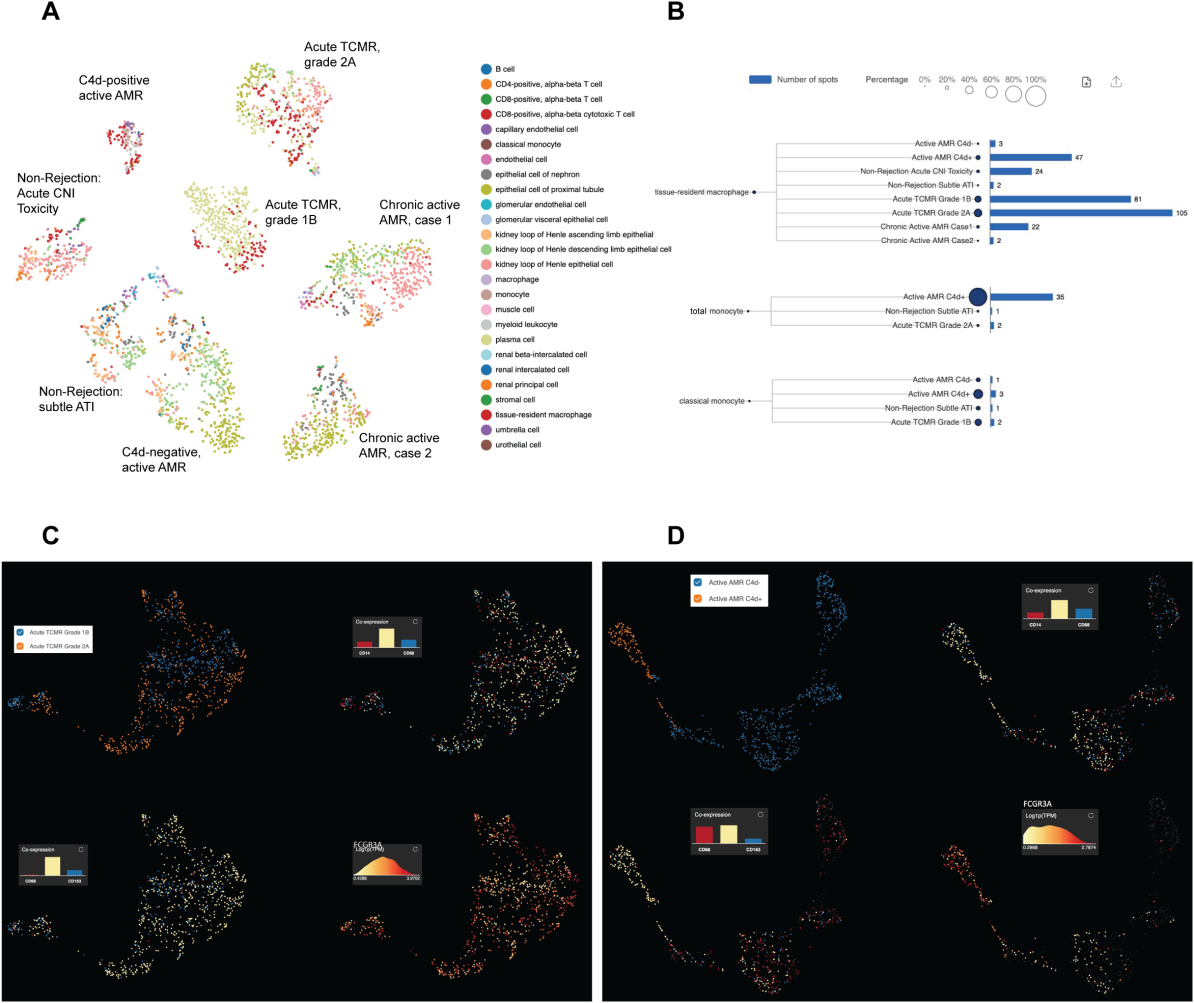
Distinct subclusters of monocytes/macrophages identified in acute rejection groups with high *FCGR3A* expression. The t-distributed stochastic neighbor embedding (t-SNE) dimension reduction and cell composition of each case was shown in **(A)**. The number of spots and percentage of macrophages, total monocytes and classical monocytes were illustrated in **(B)**. Prominent tissue-resident macrophage populations were identified in acute TCMR cases, and a significant population of non-classical and intermediate monocytes (total monocytes minus classic monocytes) was identified in C4d-positive active AMR case. UMAP analysis of acute TCMR grade 1B (blue) and grade 2A (orange) was shown in **(C)**. The clusters are overlaid with expression markers for monocytes (CD14 and CD68), macrophage (CD68 and CD163) and Fc gamma receptor IIIA (*FCGR3A*). It revealed distinct macrophage/monocytes subclusters exhibiting high expression of *FCGR3A* were evident. Similarly, UMAP analysis comparing C4d-negative (blue) and C4d-positive (orange) was shown in **(D)**. These clusters were also overlaid by monocytes and macrophage markers, as well as *FCGR3A*, which revealed distinct macrophage/monocytes subclusters with high expression of *FCGR3A*.

Acute rejection poses a significant threat to allograft survival. It is crucial to identify the specific cell populations that play key roles in various forms of acute rejection. Understanding these cellular dynamics is essential for developing potential innovative targeted therapies and improving long-term transplant outcomes. Therefore, we performed a joint visualization of spots in all cases using t-SNE dimension reduction method. The cell type composition of each case (**Figure 2A**) was generated by referencing the expression profiles of 10X Visium bins against a published meta-database of characterized kidney cells using BioTuring. Acute TCMR cases demonstrated a prominent tissue-resident macrophage population (**Figure 2B**). These tissue-resident macrophages (markers: CD68 and CD163) exhibited high expression of *FCGR3A* (**Figure 2C**). The “Monocyte category” includes classical (FCGR3A- and CD14+), intermediate monocytes (FCGR3A+ and CD14+) and non-classical monocytes (FCGR3A+ and CD14-), while the “classical monocyte” category specifically represents the classical monocytes (36). The analysis revealed that the C4d-positive active AMR case showed a significant population of non-classical and intermediate monocytes, which was the highest among and significantly different from all other cases (**Figure 2B**). This distinct subcluster of monocytes (markers: CD14 and CD68) demonstrated a high *FCGR3A* expression (**Figure 2D**). Spatial transcriptomics data analysis of *FCGR3A* expression using UMAP visualization for each case is shown in **Supplementary Figure 3**. *FCGR3A* is involved in cellular cytotoxicity and is thought to play a significant role in acute rejection (18, 37). Our findings echo those of Lamarthée et al, who demonstrated a specific association between recipient-derived *FCGR3A*+ monocytes and NK cells, and the severity of intragraft inflammation. Their study utilized different technologies - scRNA-seq and multiplexed immunofluorescence (MILAN) - on different sample types (human frozen kidney biopsy tissues).

### 3.3 Spatial distribution of monocyte/macrophage subclusters with high FCGR3A expression corresponded to the characteristic histopathological features in acute rejection groups

**Figure 3:**
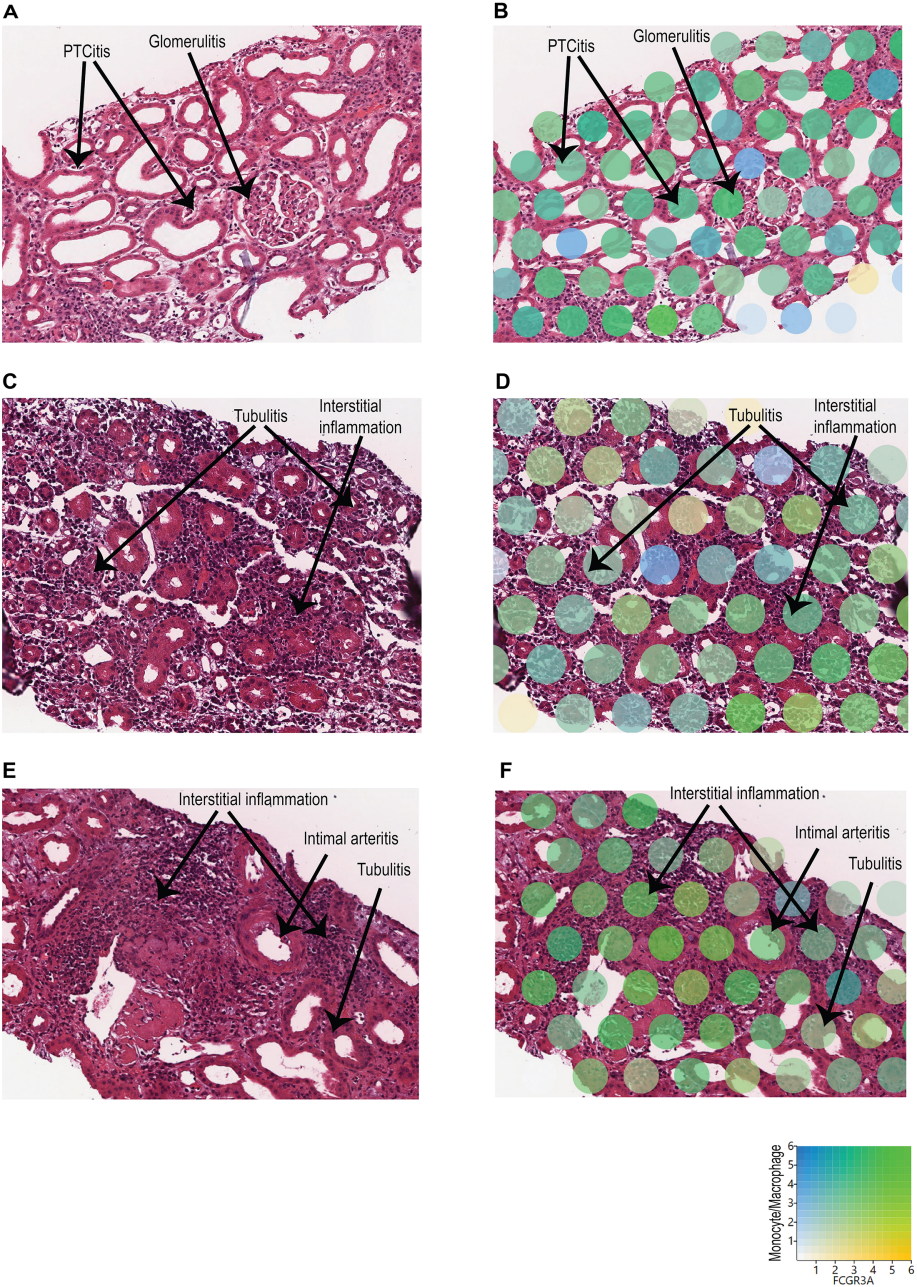
Spatial location of monocyte/macrophage subclusters with high *FCGR3A* expression. The expression of monocyte/macrophage markers (blue) and *FCGR3A* (yellow) was mapped onto the biopsy H&E images using Loupe Browser, using Log2 as scale value. Co-expression is indicated in green. **(A, B)** C4d-positive AMR: Clusters over representative areas of peritubular capillaritis (PTCitis) and glomerulitis showed enrichment in both monocyte/macrophage markers and *FCGR3A* expression. **(C, D)** Acute TCMR, grade 1B: Enrichment of monocyte/macrophage markers and *FCGR3A* expression in clusters over representative areas of tubulitis and interstitial inflammation. **(E, F)** Acute TCMR, grade 2A: Enrichment of monocyte/macrophage markers and *FCGR3A* expression in clusters over representative areas of tubulitis and interstitial inflammation. In addition, high co-expression of monocyte/macrophage markers and *FCGR3A* in inflammatory cells within the intimal arteritis (V1 lesion).

To identify the spatial locations of these distinct monocyte/macrophage subclusters, the expression of monocyte/macrophage markers and *FCGR3A* was mapped onto the biopsy H&E images using Loupe Browser (**Figure 3**). In C4d-positive AMR, clusters over representative areas of peritubular capillaritis (PTCitis) and glomerulitis showed enrichment in both monocyte/macrophage markers and *FCGR3A* expression (**Figures 3A, B**). In acute TCMR, both grade 1B (**Figures 3C, D**) and grade 2A (**Figures 3E,F**) cases demonstrated enrichment of monocyte/macrophage markers and *FCGR3A* expression in clusters over representative areas of tubulitis and interstitial inflammation. Additionally, inflammatory cells in the intimal arteritis (V1 lesion) of the acute TCMR grade 2A case exhibited high co-expression of monocyte/macrophage markers and *FCGR3A* (**Figures 3E, F**).

### 3.4 Functional pathway and gene network analysis

**Figure 4:**
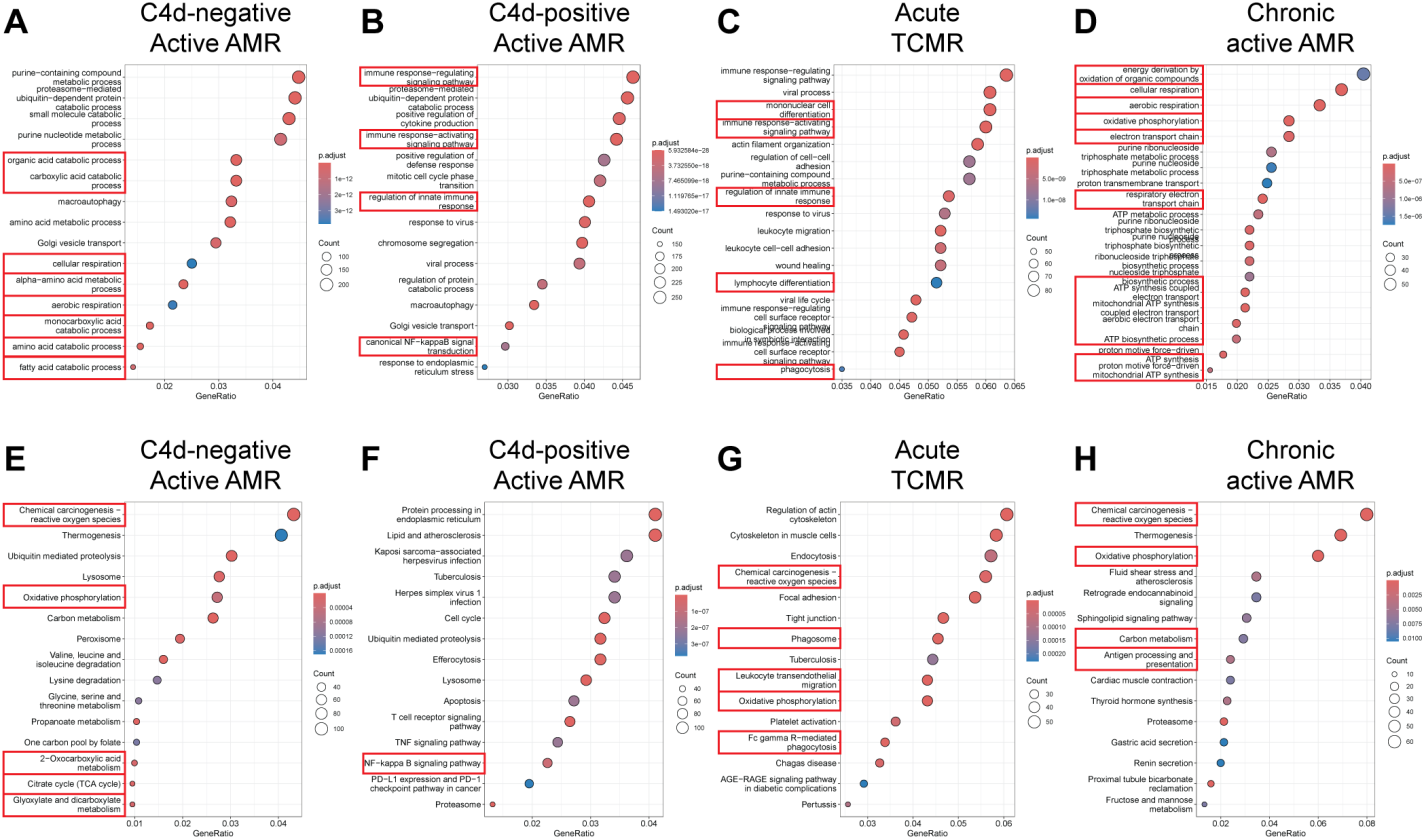
Functional pathway and gene network analysis. **(A–D)** Gene Ontology (GO) Enrichment Analysis. Key pathways (highlighted with red rectangles) associated with metabolic changes in trained immunity were upregulated in C4d-negative active AMR **(A)** and chronic active AMR **(D)**. In C4d-positive active AMR **(B)** and acute TCMR **(C)**, we observed upregulation of pathways related to activation and regulation of immune response including innate immunity. **(E–H)** Kyoto Encyclopedia of Genes and Genomes (KEGG) Analysis. Key metabolic pathways (highlighted with red rectangles) aligned with the GO analysis in both C4d-negative active AMR **(E)** and chronic active AMR **(H)**. In addition to GO analysis, KEGG analysis revealed upregulation of additional rejection-associated damage and macrophage response to transplant allografts pathways in TCMR **(G)**. It also highlighted antigen processing and presentation pathways in chronic active AMR **(H)**.

To identify enriched functional pathway associated with DEG, we performed functional pathway analysis of the DEGs using GO enrichment analysis and KEGG analysis (29). GO analysis revealed top perturbed GO biological process pathways enriched in all rejection groups, with key pathways associated with metabolic changes in trained immunity (**Figures 4A–D**). For instance, carboxylic acid catabolic, amino acid and fatty acid metabolic process pathways were upregulated in C4d-negative active AMR (**Figure 4A**). Intermediates from these process can enter glycolysis and the tricarboxylic acid (TCA) cycle, linking these pathways together (38). In chronic active AMR, there was an increase in aerobic glycolysis and mitochondrial oxidative metabolism (such as oxidative phosphorylation, respiratory electron transport chain, and Adenosine triphosphate (ATP) synthesis) (**Figure 4D**). In contrast to C4d-negative active AMR and chronic active AMR, we observed several key immune-related pathways in C4d-positive active AMR (**Figure 4B**). These included pathways involved in activating and regulating immune responses, as well as those regulating innate immune responses and NF-kappa B signaling. These findings parallelled our observations in acute TCMR, where we also identified upregulation of pathways associated with mononuclear cells (lymphocytes and monocytes/macrophages) differentiation, immune response-activating signaling pathways, phagocytosis, and regulation of innate immune response (**Figure 4C**).

KEGG analysis supported the GO analysis findings, revealing similar upregulation of metabolic pathways in both C4d-negative active AMR and chronic active AMR (**Figures 4E–H**). Moreover, both conditions exhibited increased ROS production. In addition to the immune-related pathways identified in the GO analysis, KEGG analysis uncovered upregulation of additional rejection-associated damage and macrophage response to transplant allografts pathways in TCMR, including ROS production, leukocyte trans-endothelial migration and FcγR-mediated phagocytosis (**Figure 4G**). Furthermore, KEGG analysis revealed upregulation of antigen processing and presentation pathways in chronic active AMR (**Figure 4H**).

### 3.5 Upregulation of CD47 and SIPRα in acute rejection

**Figure 5:**
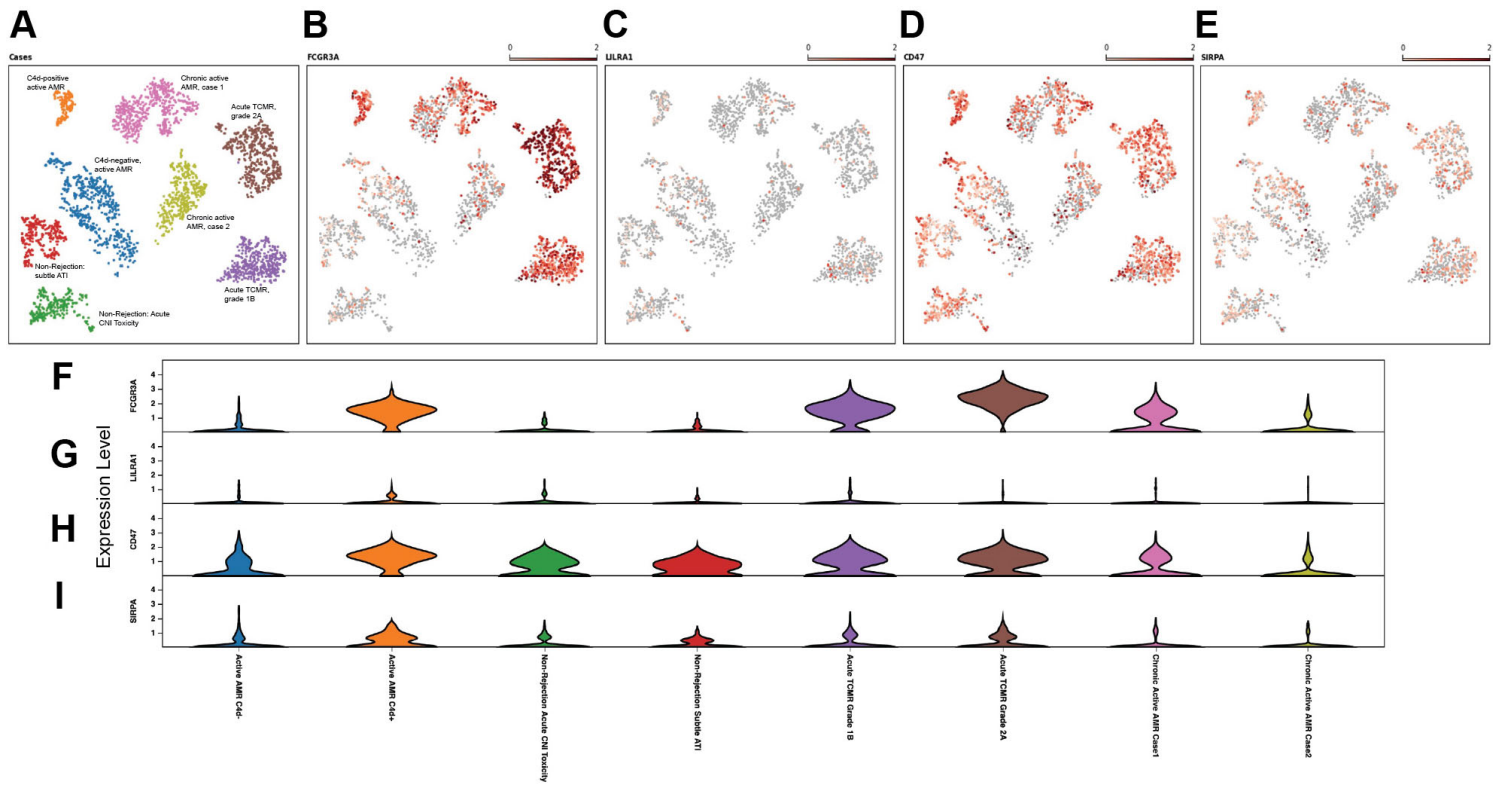
Upregulation of *CD47 and SIPRα* in acute rejection. The UMAP visualization **(A–E)**, and violin plots of log2 fold changes **(F–I)** illustrated that *CD47* expression was significant higher **(D, H)** and signal-regulatory protein-α (*SIPRα*) expression was notably higher **(E, I)** in C4d-positive active AMR case. *CD47* and *SIPRα* expression were also upregulated in acute TCMR cases, but not as pronounced as in the C4d-positive active AMR case. *FCGR3A* was significantly upregulated in both C4d-positive active AMR and acute TCMR cases **(B, F)**. However, we did not observe leukocyte immunoglobulin-like receptor A (*LILRA*) expression upregulation among these cases **(C, G)**.

Innate allorecognition, which allows innate immune cells to discriminate between self and non-self, is one of the most important mechanisms of innate immune activation during acute transplant rejection (39). CD47, leukocyte immunoglobulin-like receptor A (LILRA), and signal-regulatory protein-α (SIPRα) are key markers associated with monocytes/macrophage activation and function in both transplant rejection and trained immunity within the innate alloantigen recognition pathway (40). The LILR family consists of 11 innate immunomodulatory receptors, primarily expressed on lymphoid and myeloid cells. Based on their signaling domains, LILRs are classified as either activating (LILRA) or inhibitory (LILRB). LILRA1–2 and LILRA4-6, with the exception of the soluble LILRA3, mediate immune activation, whereas LILRB1–5 primarily inhibit immune responses and promote tolerance (41). On allograft tissues, SIPRα and MHC class I antigens are expressed and are recognized by CD47 and LILRA that are expressed on host monocytes, respectively. The UMAP visualization (**Figures 5A–E**) and violin plots of log2 fold changes (**Figures 5F–I**, **Supplementary Figures 4A–D**) illustrated significantly higher expression of *CD47* (*p*<0.05) (**Figures 5D, H**, **Supplementary Figure 4C**) and notably higher expression of *SIRPα* in C4d-positive active AMR (**Figures 5E, I**, **Supplementary Figure 4D**). *CD47* and *SIPRα* expression are also upregulated in acute TCMR cases. However, this upregulation is not as pronounced as in the C4d-positive active AMR case. The interaction between *FCGR3A* and *LILRA* is believed to play important roles in monocytes/macrophage activation and function during transplant rejection (40, 42, 43), and we observed significant upregulation of *FCGR3A* in C4d-positive active AMR and acute TCMR cases (**Figures 5B, F**, **Supplementary Figure 4A**). However, we did not observe significant *LILRA1–6* expression upregulation among these cases (**Figures 5C, G**, **Supplementary Figures 4B, E**). Although LILRB1–5 generally suppress immune responses and promote tolerance, *LILRB2* expression is notably increased in C4d-positive active AMR case. This may be explained by recent findings that LILRB2 activation is associated with macrophage recruitment and an inflammatory macrophage phenotype, as observed in non-alcoholic steatohepatitis (NASH) (44).

### 3.6 Altered metabolic genes expression related to trained immunity

**Figure 6:**
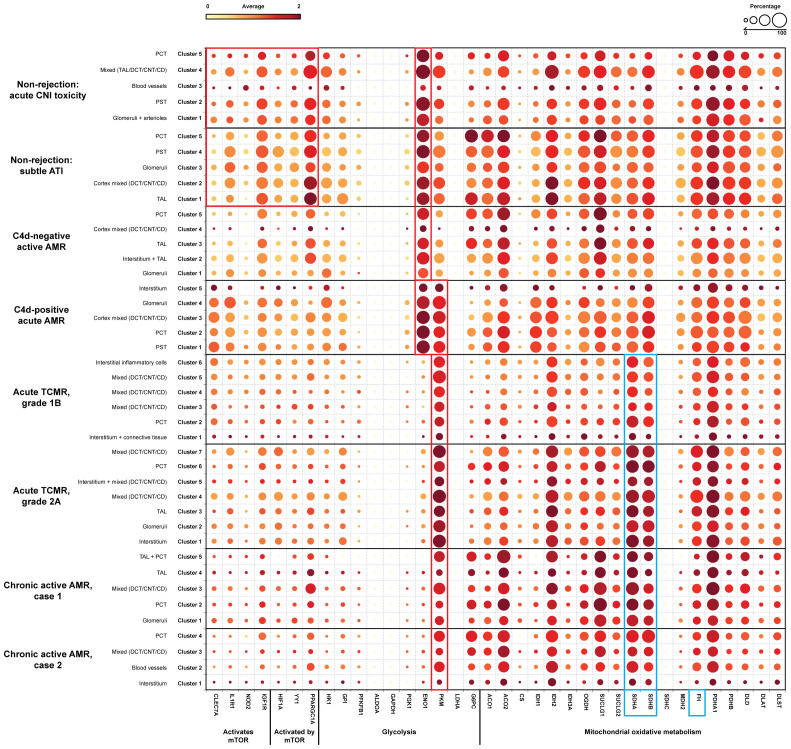
Alter gene expression of metabolic genes related to trained immunity. A dotplot analysis of metabolic genes related to trained immunity revealed distinct patterns across non-rejection and rejection conditions. Non-rejection conditions (acute CNI toxicity and subtle ATI) showed increased activity in mTOR, glycolysis, and mitochondrial oxidative metabolism, while all rejection groups exhibited more pronounced glycolytic and oxidative metabolism. Notably, enolase 1 (*ENO*1) was elevated in non-rejection conditions and C4d-negative active AMR, while pyruvate kinase (*PKM*) was significantly increased in acute TCMR and chronic active AMR (red rectangle). C4d-positive active AMR showed significant increases in both genes (red rectangle). Acute TCMR and chronic active AMR clusters also displayed elevated levels of succinate dehydrogenase A/B (*SDHA/SDHB*), metabolic intermediates associated with epigenetic changes (blue rectangle). The dotplot includes genes: 1) genes activate mTOR pathway: *CLEC7A* (C-type lectin domain family 7 member A), *IL1R1* (Interleukin 1 Receptor Type 1), *NOD2* (Nucleotide Binding Oligomerization Domain Containing 2), *IGF1R* (Insulin Like Growth Factor 1 Receptor); 2) genes activated by mTOR pathway: *HIF1A* (Hypoxia-Inducible Factor 1-alpha), *YY1* (Yin Yang 1), *PPARGC1A* (Peroxisome proliferator-activated receptor-γ coactivator 1-α); 3) glycolysis: *HK*1 (Hexokinase 1), *GPI* (Glucose-6-phosphate isomerase), *PFKFB1* (6-Phosphofructo-2-Kinase/Fructose-2,6-Biphosphatase 1), *ALDOA* (Aldolase A), *GAPDH* (Glyceraldehyde-3-phosphate dehydrogenase), *PGK1* (Phosphoglycerate kinase 1), *ENO*1, *PKM*, *LDHA* (Lactate dehydrogenase A), *G6PC* (Glucose-6-phosphatase); 4) mitochondrial oxidative metabolism: *ACO1/ACO2* (Aconitase), *CS* (Citrate synthase), *IDH1/IDH2* (Isocitrate dehydrogenase), *OGDH* (α-ketoglutarate dehydrogenase), *SUCLG1/SUCLG2* (Succinyl-CoA ligase), *SDHA/SDHB/SDHC* (Succinate dehydrogenase complex), *MDH2* (Malate dehydrogenase), *FH* (Fumarase), *PDHA1/PDHB* (Pyruvate dehydrogenase), *DLD* (Dihydrolipoamide dehydrogenase), *DLAT* (Dihydrolipoamide S-acetyltransferase), DLST (Dihydrolipoamide S-succinyltransferase) and 5) metabolic intermediates that believed to induce epigenetic changes: *FH* gene and *SDHA/SDHB* (blue rectangle).

The expression of key metabolic gene markers across different groups for trained immunity, including the key genes involved in glycolysis and mitochondrial oxidative metabolism were depicted as bubble plot (**Figure 6**). The bubble plot also included genes that encode metabolic intermediates, which are believed to induce epigenetic changes, such as fumarase (*FH*) gene and succinate dehydrogenase complex (*SDHA/SDHB/SDHC/SDHD*). This analysis revealed distinct expression patterns between groups experiencing rejection and those without rejection. Non-rejection conditions, such as acute calcineurin inhibitor (CNI) toxicity and subtle acute tubular injury (ATI), showed elevated activity in the mTOR pathway, glycolysis, and mitochondrial oxidative metabolism. In contrast, all rejection groups exhibited more pronounced elevations in glycolysis and mitochondrial oxidative metabolism activities than mTOR pathway activity. Notably, within glycolysis-related genes, Enolase 1 (*ENO*1) showed a significant increase in non-rejection conditions and C4d-negative active AMR, while Pyruvate kinase (*PKM*) was significantly elevated in acute TCMR groups and chronic active AMR. C4d-positive active AMR displayed significant increases in both genes. Additionally, clusters associated with acute TCMR and chronic active AMR showed evidence of increased levels of metabolic intermediates, *SDHA/SDHB*, which are thought to induce epigenetic changes.

## 4 Discussion

In this study, we have shown that FFPE core needle biopsy tissues are suitable for spatial transcriptomic analysis, and can uncover the transcriptomic signatures, signaling pathways, and spatially resolved immune landscapes in human kidney allograft rejection. We demonstrated that non-rejection, active AMR, acute TCMR and chronic active AMR have distinct transcriptomic features (**Figure 1**). We identified distinct subclusters of monocytes and macrophages with high *FCGR3A* expression in C4d-positive active AMR and acute TCMR, respectively (**Figure 2**). The spatial distribution of these distinct clusters corresponded to the characteristic histopathological features of active AMR and acute TCMR, respectively (**Figure 3**). Functional pathway and gene network analysis showed upregulation of key pathways that are associated with both metabolic changes in trained immunity and various immune responses, particularly those involving innate immunity (**Figure 4**). Moreover, key markers associated with monocytes/macrophage activation and function in both transplant rejection and trained immunity within the innate alloantigen recognition pathway showed significantly increased *CD47* and notably increased *SIPRα* in the C4d-positive active AMR case, while being less prominent in acute TCMR cases (**Figure 5**). Finally, our study revealed that the metabolic markers associated with trained innate immunity exhibited distinct expression patterns in groups experiencing rejection compared to those without rejection (**Figure 6**). These findings are summarized in **Supplementary Figure 5**. This was the first report of using spatial transcriptomics to evaluate different rejection types of FFPE core needle biopsies from human kidney allografts. Our findings complement the transcript signatures identified through bulk transcriptome microarrays, while also providing additional valuable spatial information.

Bulk transcriptomic microarrays, such as MMDX, have been applied to assist in the clinical diagnosis of rejection. However, these methods typically require relatively large tissue volumes, which are challenging to obtain through core needle biopsies. Moreover, these techniques extract analytes from tissue and sequence them in bulk. Data regarding the type of cells expressing a given transcript, the location of these cells within the tissue, and co-expression of transcripts in the tissue geography are all lost by this bulk preparation. Single cell RNA sequencing (scRNA-seq) is a recently developed technology exclusively used in research to analyze gene expression at the individual cell level. While it offers valuable insights into cellular heterogeneity, it has limitations: it typically requires fresh or frozen tissue samples, necessitates a high number of isolated cells that are hard to obtain by core needle biopsy tissue, and loses spatial information. Our approach of using spatial transcriptomics to evaluate rejection on archived FFPE core needle biopsies from human allografts has the potential to bridge the gap between histopathologic and molecular classifications. This approach likely provides more comprehensive information while requiring only minimal tissue input.

Despite advances in immunosuppression regimens used in solid organ transplantation over the past decades, achieving long-term success has been hindered by several challenges, including the need to tailor post-transplant immunosuppression regimens to ensure patient-specific optimization (45). Current immunosuppressive treatment regimens only target adaptive immune cells. There is a lack of potential biomarkers for innovative immunosuppressive therapies. Although research in the field of innate immunity in transplant immunology has garnered attention in recent years, there is a limited knowledge of the specific transcript signatures associated with innate immune cells during post-transplant events. These events include non-rejection conditions (such as subclinical graft injury, delayed graft function, ATI, CNI toxicity and inflammation below diagnostic thresholds for rejection), early acute rejection, and chronic rejection. Of particular interest are monocytes/macrophages and NK cells, which play critical roles in the innate immune response to transplant allografts by producing proinflammatory factors, killing graft cells, and enhancing the adaptive immune response (4–8). Furthermore, organ transplantation induces trained innate immunity, contributing to allograft rejection. However, large knowledge gaps persist regarding their molecular and cellular mechanism, duration, adaptability and impact on adaptive immunity in human organ transplantation. While clinical trials are ongoing, current immunosuppressive treatment regimens still fail to leverage the potential benefits of modulating the innate immune response. There is an urgent need to discover potential biomarkers for future innovative immunosuppressive therapies. Our discovery of distinct monocytes/macrophages subclusters based on spatial transcriptomics and the associated signaling pathways in acute rejection, can uncover potential biomarkers, such as *FCGR3A*, for future novel immunosuppressive therapy targets. Notably, polymorphisms in FcγRIIIA (158V/F) have been demonstrated to enhance NK cell affinity for IgG and increase risk of graft failure. Furthermore, the 158 V/V genotype specifically has been linked to decreased survival rates in renal allografts with chronic active AMR (46–50).

An unexpected but potentially important finding was that the C4d-positive active AMR case had significantly different spatial transcriptomic features than the C4d-negative active AMR case. Gupta et al. found no differences in gene expression between C4d positive and C4d negative biopsies with MVI >2 using microarrays (51). Our results suggested that spatial transcriptomics may offer a potential advantage over microarray analysis in identifying distinct molecular signatures associated with different morphologic subsets of AMR. However, we understand that our study was limited by having only one case each of C4d-positive and C4d-negative active AMR. We are currently planning a study with a larger sample size to compare these two conditions, which should yield more robust and representative results in the future.

We acknowledge the limitations of our current study. First, the fixed 55-μm diameter map spots on the transcriptomic platform resulted in variable cell densities associated with each barcode. This constraint may have introduced analytical inconsistencies between samples and potentially caused us to overlook less prominent subclusters, such as NK cells. We cannot exclude that the upregulated expression of *FCGR3A* was in part derived from NK cells. In future experiments, this technical limitation could likely be addressed by applying the newly developed 10x Genomics Visium high definition (HD) or Xenium *In Situ* spatial transcriptomics platforms. Secondly, our study is limited by the number of map spots in capture areas (6 mm x 6 mm). This limitation is due to the nature of kidney needle core biopsy tissue, which is typically small, and the empty gaps between individual tissue cores within the paraffin blocks. To overcome this issue in future studies, we could use larger capture areas (1 cm x 1 cm) and carefully select cases with multiple needle cores.

In summary, our study demonstrated that the non-rejection, active AMR, acute TCMR and chronic active AMR exhibited distinct spatial transcriptomic features. Our discovery of the unique monocyte/macrophage subclusters with high *FCGR3A* expression may shed light on the mechanism underlying acute kidney rejection and reveal potential cellular targets for innovative immunosuppressive therapies.

## Data Availability

The data that supports the findings of this study are publicly available in the GEO, accession # GSE280559, with the following link: https://www.ncbi.nlm.nih.gov/geo/query/acc.cgi?acc=GSE304669.
