# Supplementary material for: Spatial transcriptomics reveals distinct role of monocytes/macrophages with high FCGR3A expression in kidney transplant rejections
Source: Front Immunol. 2025 Sep 15;16:1654741. doi: 10.3389/fimmu.2025.1654741 (PMC12477047; doi:10.3389/fimmu.2025.1654741)
Supplement: Supplementary file 1 [file DataSheet1.docx]

Supplementary Material

# Supplementary Figures and Tables

## Supplementary Figures

**
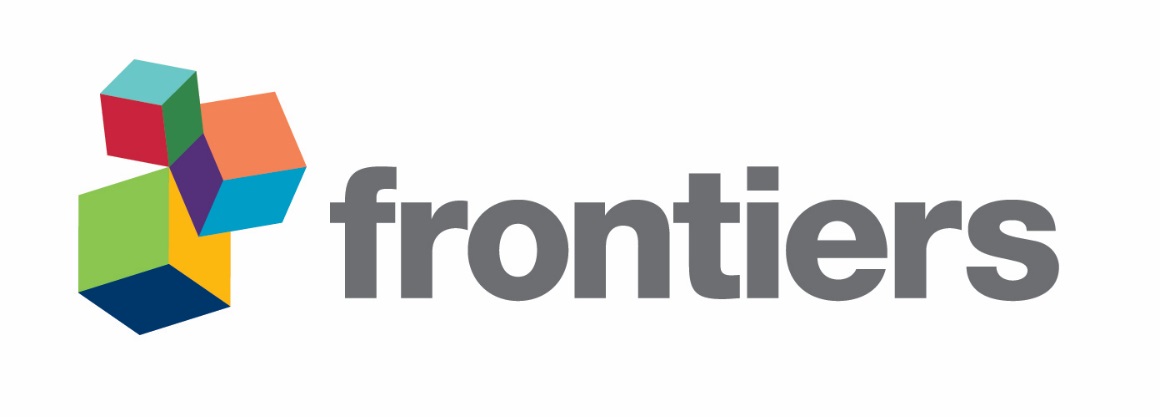
**

**Supplementary Figure 1.** **Spatial transcriptomics can be successfully performed on FFPE core needle biopsies of human kidney allografts.** Transcriptomic map composed of “spots” (55 μm in diameter) with unique RNA expression signatures. The spots were localized on the kidney histologic image with barcodes. Four to seven unsupervised clusters in each case were shown by segmentation analysis and visualized by Uniform manifold approximation and projection (UMAP) algorithm. The cluster annotation was based on histopathologic features and known gene expression markers associated with kidney structures. Due to the size of the spots employed in spatial transcriptomics, the cell densities associated with each barcode are variable, ranging from 1 to 10 per spot. As a result, some clusters showed admixed contribution of different cellular markers, such as tubular markers. CNI: acute calcineurin inhibitors; ATI: toxicity acute tubular injury; AMR: active antibody mediated rejection; TCMR: cell mediated rejection; PST: proximal straight tubule; PCT: proximal convoluted tubules; TAL: thick ascending limb; DCT: distal convoluted tubules; CNT: connecting tubules; CD: collecting duct.

**Supplementary Figure 2. Overlap and distinctions between FFPE tissue transcriptomic signatures and published RNA signatures from bulk transcriptome microarrays.** The Venn diagram illustrated the overlap and distinctions between our identified FFPE tissue transcriptomic signatures of active AMR (A), acute TCMR (B), chronic active AMR (C) and previously published top 30 transcripts associated with AMR-selected, TCMR-selected and universal rejection by MMDX (Halloran *et al*. 2017 paper). In addition, Venn diagram illustrated the overlap and distinctions between our identified FFPE tissue transcriptomic signatures of active AMR (D), acute TCMR (E), and chronic active AMR (F) and previously published top 20 transcripts associated AMR, TCMR, and injury- and rejection-associated transcript by MMDX (Halloran *et al*. 2024 paper), respectively.

**Supplementary Figure 3: Fc gamma receptor IIIA (*FCGR3A*) expression of each case using Uniform manifold approximation and projection (UMAP) visualization**. *FCGR3A* was significantly upregulated in both C4d-positive active AMR and acute TCMR cases, while non-rejection cases and C4d-negative active AMR exhibited very low expression levels. Chronic active AMR cases showed intermediate expression levels. The color intensity in the visualization represents *FCGR3A* gene expression levels, with red shades indicating higher expression and yellow shades indicating lower expression**.** CNI: acute calcineurin inhibitors; ATI: toxicity acute tubular injury.

**Supplementary Figure 4: Upregulation of *CD47 and* *SIPRα* in acute rejection**. The violin plots of log2 fold changes illustrated that *CD47* expression was significant higher **(C)** and signal-regulatory protein-α (*SIPRα*) expression was notably higher **(D)** in C4d-positive active AMR case. *CD47* and *SIPRα* expression were also upregulated in acute TCMR cases, but not as pronounced as in the C4d-positive active AMR case. *FCGR3A* was significantly upregulated in both C4d-positive active AMR and acute TCMR cases **(A)**. However, we did not observe leukocyte immunoglobulin-like receptor A (*LILRA*) expression upregulation among these cases **(B)**. The box within each violin plot represents the interquartile range of the data, with the mean represented as a white dot.

**Supplementary Figure 5**: **Integration of histopathology, pathological diagnosis, and spatial transcriptomics in eight kidney transplant cases**. This figure presents a comprehensive analysis of these kidney transplant cases, illustrating the progression from histopathological features to pathological diagnosis and spatial transcriptomic analysis. The pathological diagnoses include acute CNI toxicity (characterized by isometric vacuolization), active AMR (featuring peritubular capillaritis and glomerulitis, subclassified into C4d-positive and C4d-negative variants), acute TCMR (characterized by tubulitis and interstitial inflammation), and chronic active AMR (featuring transplant glomerulopathy and transplant arteriopathy). The figure demonstrates how these distinct histopathological features inform the pathological diagnosis and how spatial transcriptomics provide additional molecular insights into each condition, offering a multi-layered view of kidney transplant pathology.
