# Supplementary material for: Spatial transcriptomics reveals distinct role of monocytes/macrophages with high FCGR3A expression in kidney transplant rejections
Source: Front Immunol. 2025 Sep 15;16:1654741. doi: 10.3389/fimmu.2025.1654741 (PMC12477047; doi:10.3389/fimmu.2025.1654741)
Supplement: Supplementary file 2 [file Table1.docx]

**Supplementary Table 1: Top 30 genes (sorted by Log2 fold change (Log2FC) value) selectively increased in biopsies with active AMR, acute TCMR and chronic active AMR**

| **Active AMR, C4d-** | | | **Active AMR, C4d+** | | | **Acute TCMR, grade 1B** | | | **Acute TCMR, grade 2A** | | | **Chronic active AMR, case #1** | | | **Chronic active AMR, case #2** | | |
| --- | --- | --- | --- | --- | --- | --- | --- | --- | --- | --- | --- | --- | --- | --- | --- | --- | --- |
| Genes | p-value | Log2FC | Genes | p-value | Log2 FC | Genes | p-value | Log2 FC | Genes | p-value | Log2 FC | Genes | p-value | Log2 FC | Genes | p-value | Log2 FC |
| AGMAT | 5.0E-131 | 2.91 | AKR1B10 | 3.4E-64 | 5.34 | IGLC1 | 2.4E-228 | 3.96 | APOC1 | 3.5E-147 | 3.81 | PAPPA2 | 1.1E-78 | 2.69 | CALML3 | 4.6E-44 | 3.60 |
| CTXN3 | 1.4E-73 | 2.78 | **S100A8** | 2.9E-88 | 4.67 | DERL3 | 2.6E-131 | 3.9 | LTF | 3.8E-205 | 3.69 | IFITM10 | 3.5E-43 | 2.54 | EPO | 1.3E-03 | 2.80 |
| SMIM24 | 2.7E-202 | 2.7 | UBD | 2.2E-82 | 4.57 | IGKC | 3.9E-252 | 3.65 | COL1A1 | 1.9E-265 | 3.63 | PLCH2 | 6.2E-29 | 2.42 | APOC3 | 6.1E-33 | 2.62 |
| CYP2B6 | 3.7E-22 | 2.69 | AKR1C3 | 1.9E-69 | 4.46 | MZB1 | 2.5E-192 | 3.56 | NNMT | 1.9E-200 | 3.28 | AMN | 1.7E-178 | 2.40 | CYP17A1 | 2.9E-52 | 2.56 |
| SORD | 8.9E-68 | 2.63 | **S100A9** | 2.8E-93 | 4.34 | SLPI | 1.1E-166 | 3.41 | SERPINE1 | 1.8E-146 | 3.08 | IRX1 | 3.1E-52 | 2.35 | PRAP1 | 1.8E-101 | 2.51 |
| SLC34A1 | 2.4E-115 | 2.45 | HSPA5 | 3.8E-69 | 4.25 | JCHAIN | 3.7E-241 | 3.24 | C1QC | 3.1E-267 | 3.07 | CNTNAP3B | 4.3E-04 | 2.32 | MT1H | 1.5E-89 | 2.46 |
| ATP5IF1 | 8.8E-76 | 2.44 | MT2A | 2.6E-90 | 4.09 | **IRF4** | 2.2E-101 | 3.05 | **FCGR3A** | 6.5E-224 | 2.97 | KLK1 | 2.3E-13 | 2.23 | RBP4 | 9.9E-56 | 2.29 |
| SLC36A2 | 5.9E-74 | 2.41 | NQO1 | 5.7E-78 | 3.98 | ZBP1 | 6.7E-53 | 3.03 | IGHM | 4.0E-199 | 2.94 | GP2 | 7.5E-53 | 2.18 | FGB | 1.3E-58 | 2.27 |
| MRLN | 4.0E-07 | 2.4 | MT1G | 5.3E-59 | 3.94 | IGHG1 | 2.0E-215 | 2.74 | C1QA | 1.9E-257 | 2.92 | CLCNKA | 1.2E-49 | 2.13 | C2ORF54 | 4.8E-09 | 2.24 |
| CCL15 | 2.1E-20 | 2.39 | TYROBP | 2.1E-76 | 3.67 | PIM2 | 1.6E-123 | 2.71 | C1QB | 1.3E-253 | 2.87 | WNK4 | 1.0E-65 | 2.10 | AOC1 | 7.6E-110 | 2.23 |
| FAM151A | 7.7E-55 | 2.38 | CYP4F11 | 2.9E-45 | 3.57 | ANGPTL4 | 1.5E-37 | 2.68 | CD163 | 5.2E-219 | 2.78 | MROH7 | 1.5E-29 | 1.99 | SERPINA5 | 7.4E-66 | 2.18 |
| MT1X | 3.7E-111 | 2.35 | PDIA4 | 6.2E-51 | 3.48 | XBP1 | 8.0E-178 | 2.64 | FN1 | 9.9E-218 | 2.73 | SLC9A3 | 1.3E-85 | 1.90 | SCNN1G | 5.6E-34 | 1.97 |
| MDH1 | 5.3E-54 | 2.35 | CCL13 | 6.3E-29 | 3.48 | TXNDC5 | 1.4E-213 | 2.62 | **C3** | 3.4E-165 | 2.62 | C16ORF89 | 8.4E-34 | 1.87 | VTN | 3.5E-05 | 1.93 |
| HBA2 | 1.8E-04 | 2.31 | GNG5 | 8.6E-13 | 3.4 | CD79A | 3.7E-61 | 2.59 | COL1A2 | 7.8E-221 | 2.61 | CLCNKB | 2.7E-37 | 1.85 | NCAM2 | 1.6E-14 | 1.86 |
| NDUFS8 | 1.2E-28 | 2.28 | GCLM | 4.3E-58 | 3.3 | BLK | 7.3E-27 | 2.56 | CLDN1 | 5.5E-99 | 2.56 | FMO4 | 7.3E-95 | 1.84 | TRIM50 | 2.2E-06 | 1.85 |
| PCK1 | 2.7E-101 | 2.28 | PRDX1 | 4.6E-70 | 3.26 | FCRL5 | 1.3E-56 | 2.56 | VSIG4 | 3.4E-164 | 2.56 | ACPP | 1.2E-46 | 1.82 | C2ORF40 | 1.9E-27 | 1.85 |
| GATM | 7.0E-183 | 2.24 | HIST1H2BB | 1.1E-10 | 3.25 | PDK1 | 5.7E-112 | 2.55 | IFITM3 | 2.4E-247 | 2.42 | AGXT | 4.0E-08 | 1.82 | GSTA1 | 7.6E-93 | 1.83 |
| C1ORF35 | 9.8E-28 | 2.23 | FCER1G | 1.1E-68 | 3.19 | SPAG4 | 2.6E-32 | 2.53 | SLC1A3 | 1.0E-67 | 2.38 | HSPA1A | 8.7E-97 | 1.78 | RHCG | 5.2E-33 | 1.83 |
| MIOX | 9.8E-135 | 2.22 | ATP5PO | 2.9E-07 | 3.08 | TNFRSF17 | 5.5E-44 | 2.52 | IFITM1 | 4.4E-105 | 2.33 | AQP7 | 2.5E-57 | 1.76 | PCDH10 | 1.1E-21 | 1.82 |
| XYLB | 9.1E-30 | 2.21 | NOP10 | 1.9E-28 | 3 | LAX1 | 9.3E-46 | 2.5 | MS4A4A | 2.7E-109 | 2.32 | ETV4 | 4.2E-21 | 1.73 | TLDC2 | 4.3E-06 | 1.80 |
| PTMS | 1.4E-44 | 2.19 | CYBB | 2.8E-75 | 2.99 | ZNF215 | 2.5E-31 | 2.42 | ADAMTS2 | 8.4E-54 | 2.31 | ACSL6 | 5.0E-12 | 1.71 | LEAP2 | 6.0E-08 | 1.80 |
| SLC34A3 | 1.6E-29 | 2.18 | GMFG | 1.4E-25 | 2.98 | MS4A1 | 2.2E-39 | 2.38 | FCGR1B | 3.1E-78 | 2.31 | EFHD1 | 1.0E-74 | 1.68 | BSG | 1.2E-131 | 1.79 |
| TMBIM6 | 9.7E-16 | 2.17 | CALR | 9.7E-71 | 2.95 | PRDM1 | 1.7E-79 | 2.36 | CDH6 | 4.9E-110 | 2.30 | GPT | 3.0E-51 | 1.67 | TRIM6 | 8.9E-44 | 1.78 |
| FMO1 | 3.5E-68 | 2.12 | HINT1 | 9.2E-41 | 2.93 | TENT5C | 9.5E-108 | 2.34 | MVP | 6.3E-138 | 2.28 | EPHB3 | 1.4E-25 | 1.67 | FABP1 | 3.9E-58 | 1.78 |
| UQCRB | 1.2E-69 | 2.09 | MARCO | 1.0E-28 | 2.89 | POU2AF1 | 4.2E-46 | 2.31 | LCN2 | 3.1E-24 | 2.23 | CAPN12 | 1.8E-27 | 1.65 | CTSV | 1.3E-12 | 1.76 |
| EMCN | 5.4E-20 | 2.09 | VNN1 | 1.8E-38 | 2.88 | IGHA1 | 2.9E-161 | 2.28 | CCL4L2 | 1.1E-39 | 2.21 | AL845331 | 2.8E-19 | 1.64 | GRP | 2.7E-02 | 1.75 |
| ZNF787 | 4.4E-06 | 2.07 | EIF1 | 5.9E-67 | 2.87 | FPR1 | 9.7E-17 | 2.27 | CXCL11 | 6.8E-110 | 2.19 | GHDC | 1.3E-56 | 1.62 | GSTA2 | 1.6E-23 | 1.73 |
| C3ORF85 | 2.3E-06 | 2.06 | NDUFB4 | 1.2E-29 | 2.87 | ANKDD1A | 3.2E-14 | 2.17 | CXCL13 | 2.7E-45 | 2.18 | GGACT | 7.4E-53 | 1.61 | OLR1 | 4.1E-16 | 1.67 |
| HBB | 5.7E-10 | 2.05 | PSMA4 | 3.0E-55 | 2.87 | ZNF534 | 1.9E-09 | 2.14 | FKBP5 | 4.9E-132 | 2.17 | MFSD3 | 2.5E-50 | 1.61 | PRELP | 1.7E-17 | 1.67 |

The genes highlighted in bold are those that play a role in acute rejection but were not listed among the top transcripts in the MMDX study.
