# Supplementary material for: Spatial transcriptomics reveals distinct role of monocytes/macrophages with high FCGR3A expression in kidney transplant rejections
Source: Front Immunol. 2025 Sep 15;16:1654741. doi: 10.3389/fimmu.2025.1654741 (PMC12477047; doi:10.3389/fimmu.2025.1654741)
Supplement: Supplementary file 3 [file Image1.pdf]

Figure 1

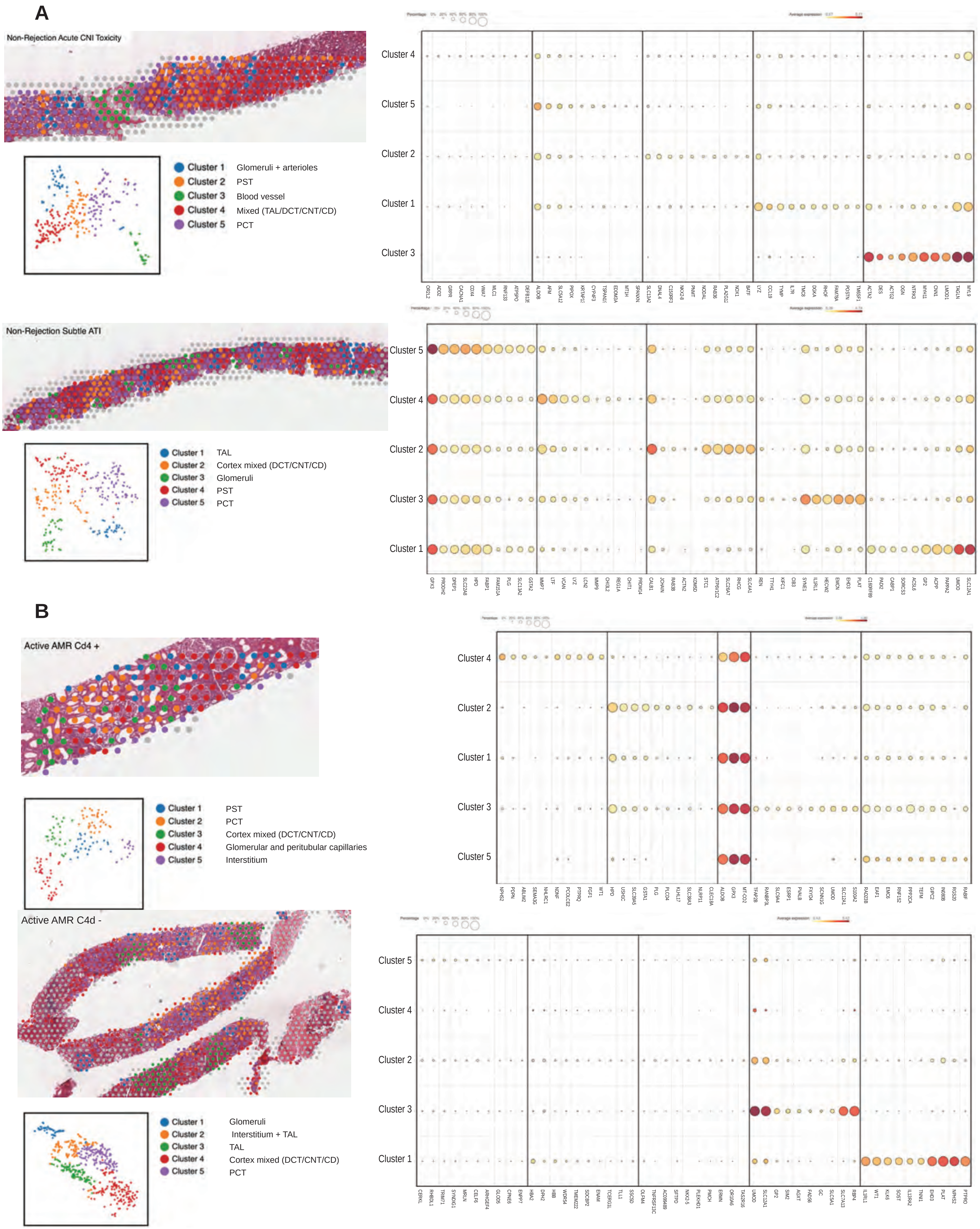
A
Non-Rejection Acute CNI Toxicity
Cluster 1 Glomeruli + arterioles
Cluster 2 PST
Cluster 3 Blood vessel
Cluster 4 Mixed (TAL/DCT/CNT/CD)
Cluster 5 PCT
Cluster 4
Cluster 5
Cluster 2
Cluster 1
Cluster 3
Non-Rejection Subtle ATI
Cluster 1 TAL
Cluster 2 Cortex mixed (DCT/CNT/CD)
Cluster 3 Glomeruli
Cluster 4 PST
Cluster 5 PCT
Cluster 5
Cluster 4
Cluster 2
Cluster 3
Cluster 1
B
Active AMR Cd4 +
Cluster 1 PST
Cluster 2 PCT
Cluster 3 Cortex mixed (DCT/CNT/CD)
Cluster 4 Glomerular and peritubular capillaries
Cluster 5 Interstitium
Cluster 4
Cluster 2
Cluster 1
Cluster 3
Cluster 5
Active AMR C4d -
Cluster 1 Glomeruli
Cluster 2 Interstitium + TAL
Cluster 3 TAL
Cluster 4 Cortex mixed (DCT/CNT/CD)
Cluster 5 PCT
Cluster 5
Cluster 4
Cluster 2
Cluster 3
Cluster 1

C

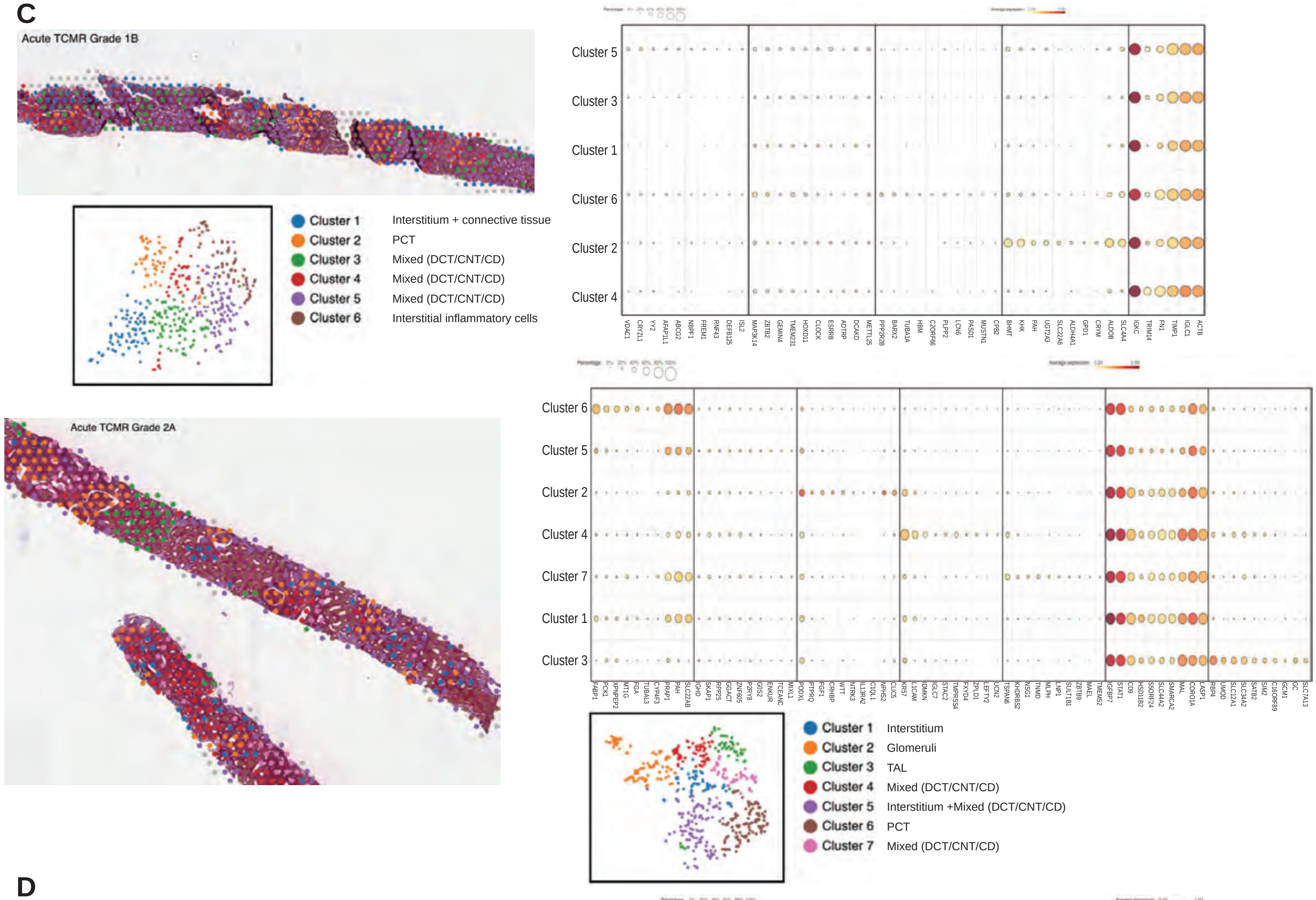
Acute TCMR Grade 1B
Cluster 1 Interstitium + connective tissue
Cluster 2 PCT
Cluster 3 Mixed (DCT/CNT/CD)
Cluster 4 Mixed (DCT/CNT/CD)
Cluster 5 Mixed (DCT/CNT/CD)
Cluster 6 Interstitial inflammatory cells
Cluster 5
Cluster 3
Cluster 1
Cluster 6
Cluster 2
Cluster 4
Acute TCMR Grade 2A
Cluster 6
Cluster 5
Cluster 2
Cluster 4
Cluster 7
Cluster 1
Cluster 3
Cluster 1 Interstitium
Cluster 2 Glomeruli
Cluster 3 TAL
Cluster 4 Mixed (DCT/CNT/CD)
Cluster 5 Interstitium +Mixed (DCT/CNT/CD)
Cluster 6 PCT
Cluster 7 Mixed (DCT/CNT/CD)

D

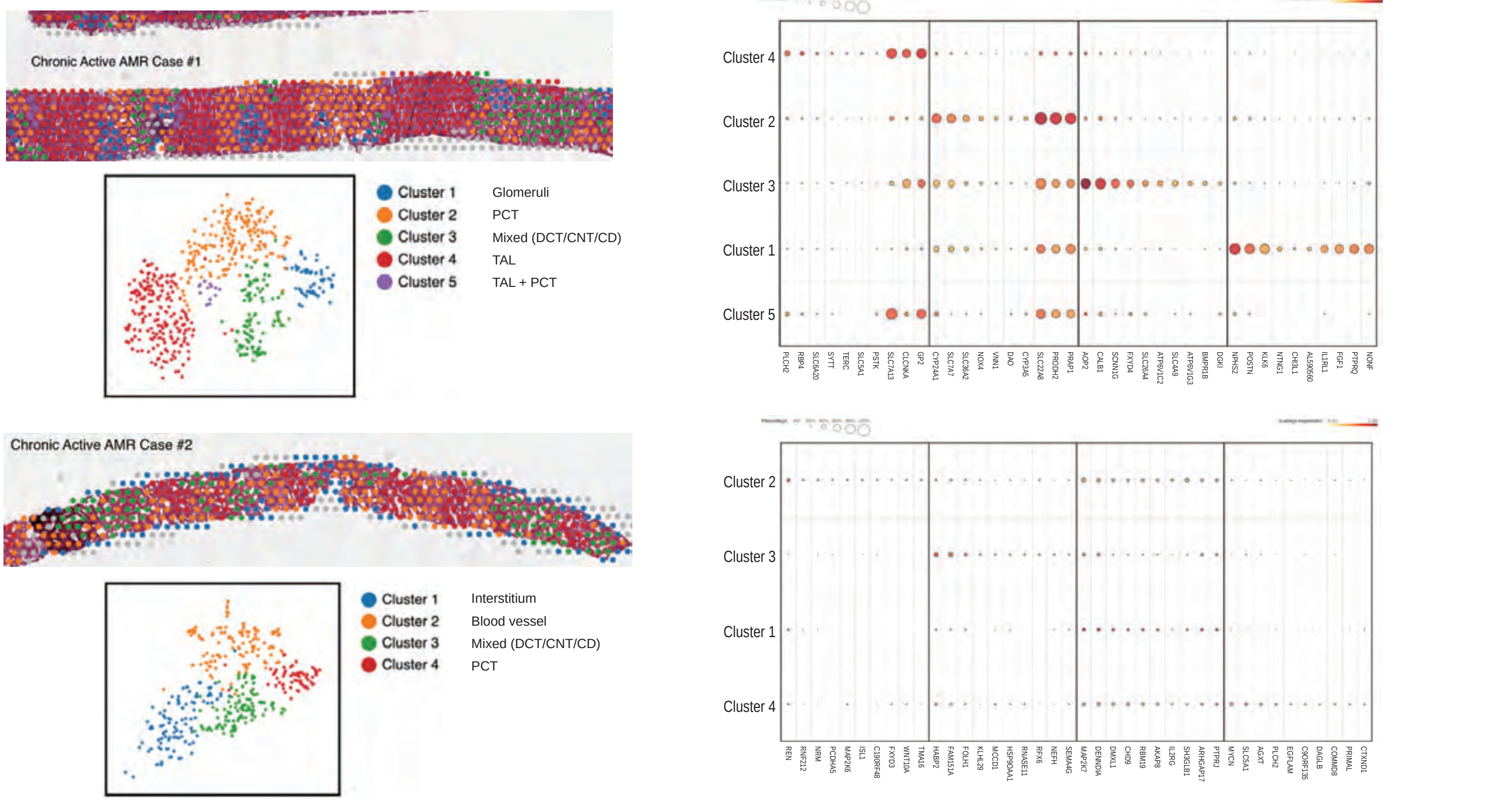
Chronic Active AMR Case #1
Cluster 1 Glomeruli
Cluster 2 PCT
Cluster 3 Mixed (DCT/CNT/CD)
Cluster 4 TAL
Cluster 5 TAL + PCT
Cluster 4
Cluster 2
Cluster 3
Cluster 1
Cluster 5
Chronic Active AMR Case #2
Cluster 1 Interstitium
Cluster 2 Blood vessel
Cluster 3 Mixed (DCT/CNT/CD)
Cluster 4 PCT
Cluster 2
Cluster 3
Cluster 1
Cluster 4
