# Supplementary figures and images for: Spatial transcriptomics reveals distinct role of monocytes/macrophages with high FCGR3A expression in kidney transplant rejections
Source: Front Immunol. 2025 Sep 15;16:1654741. doi: 10.3389/fimmu.2025.1654741 (PMC12477047; doi:10.3389/fimmu.2025.1654741)

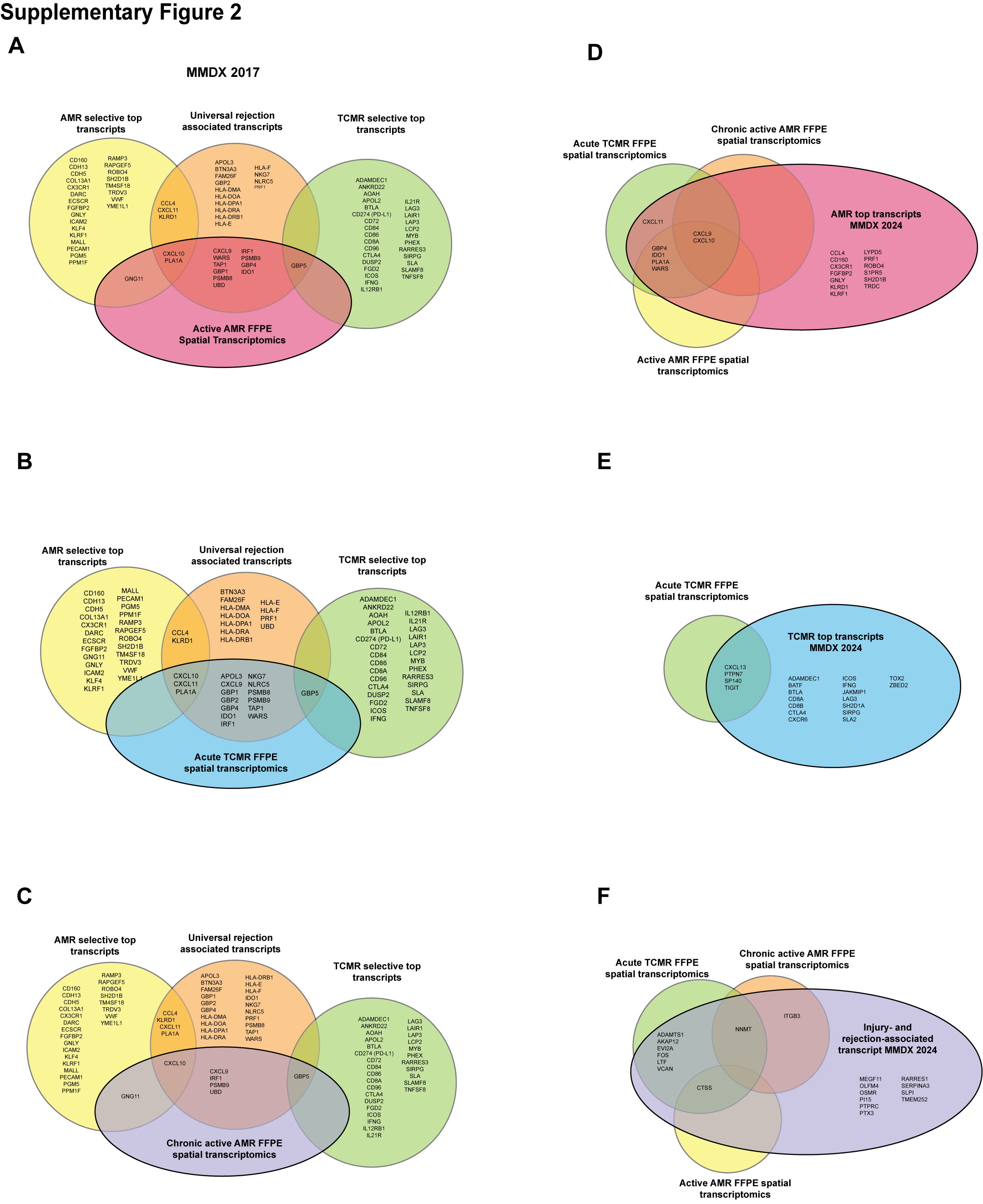

Supplement: Supplementary file 4 [file Image2.tif]

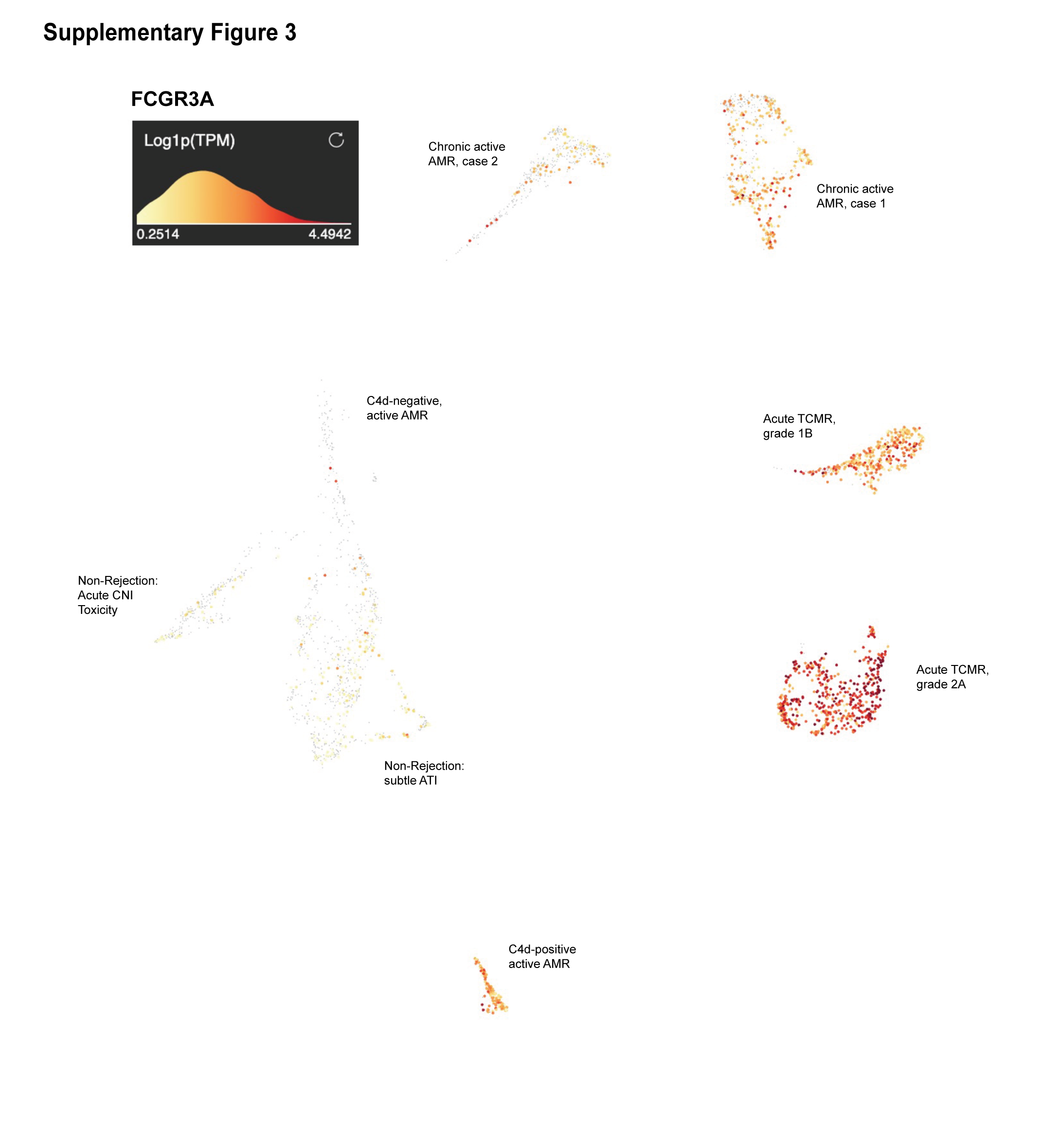

Supplement: Supplementary file 5 [file Image3.tif]

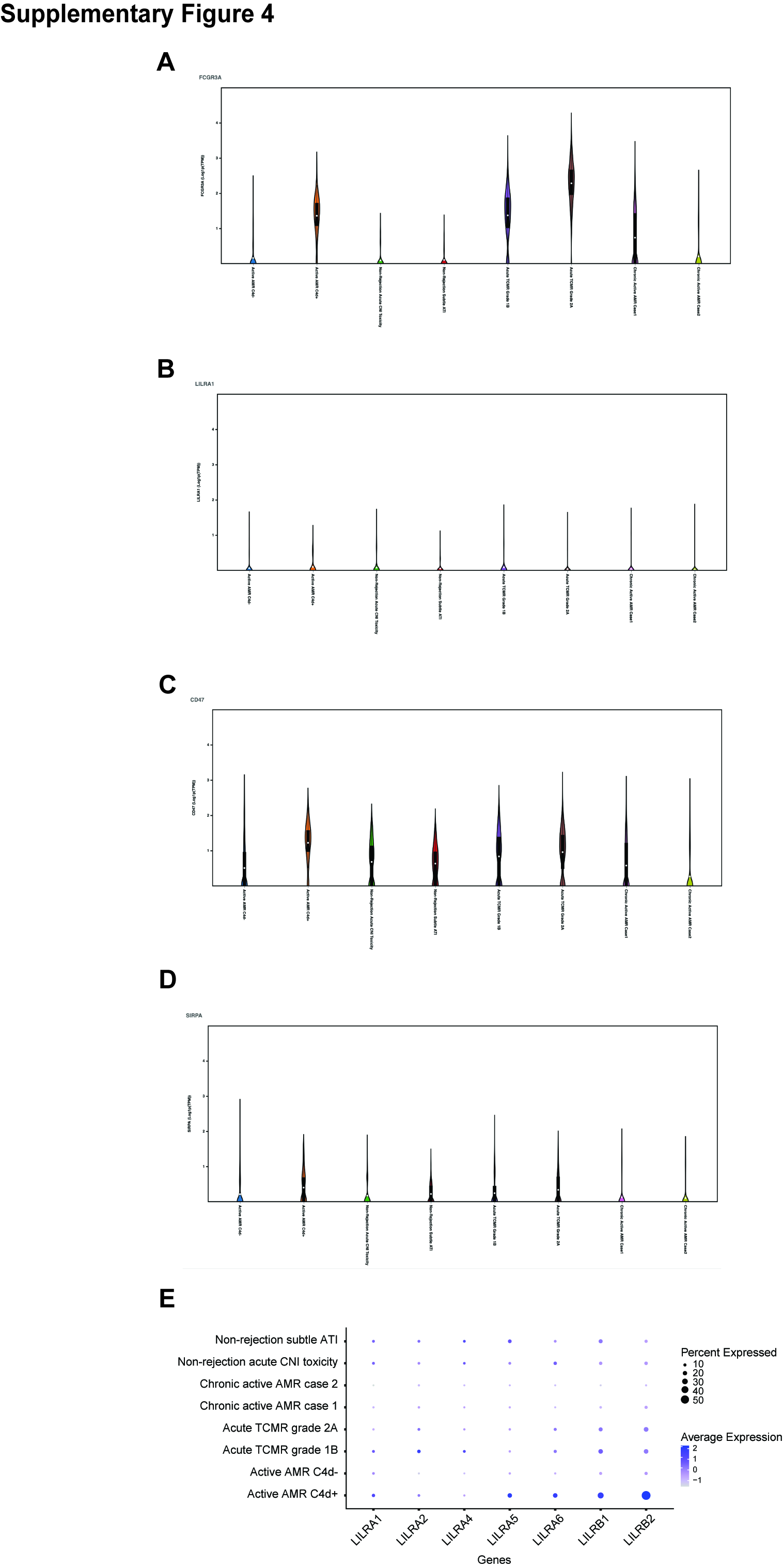

Supplement: Supplementary file 6 [file Image4.tif]

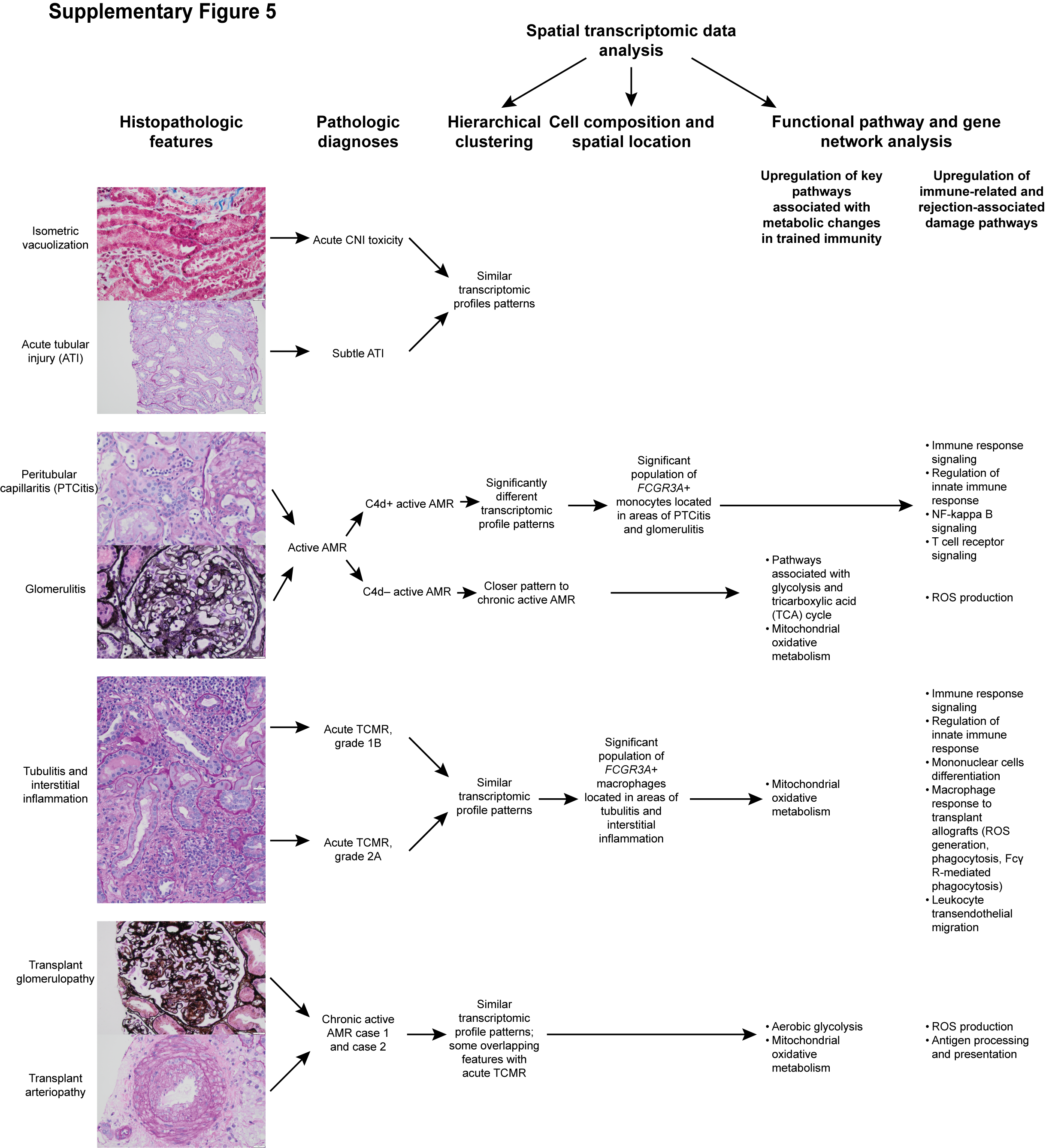

Supplement: Supplementary file 7 [file Image5.tif]

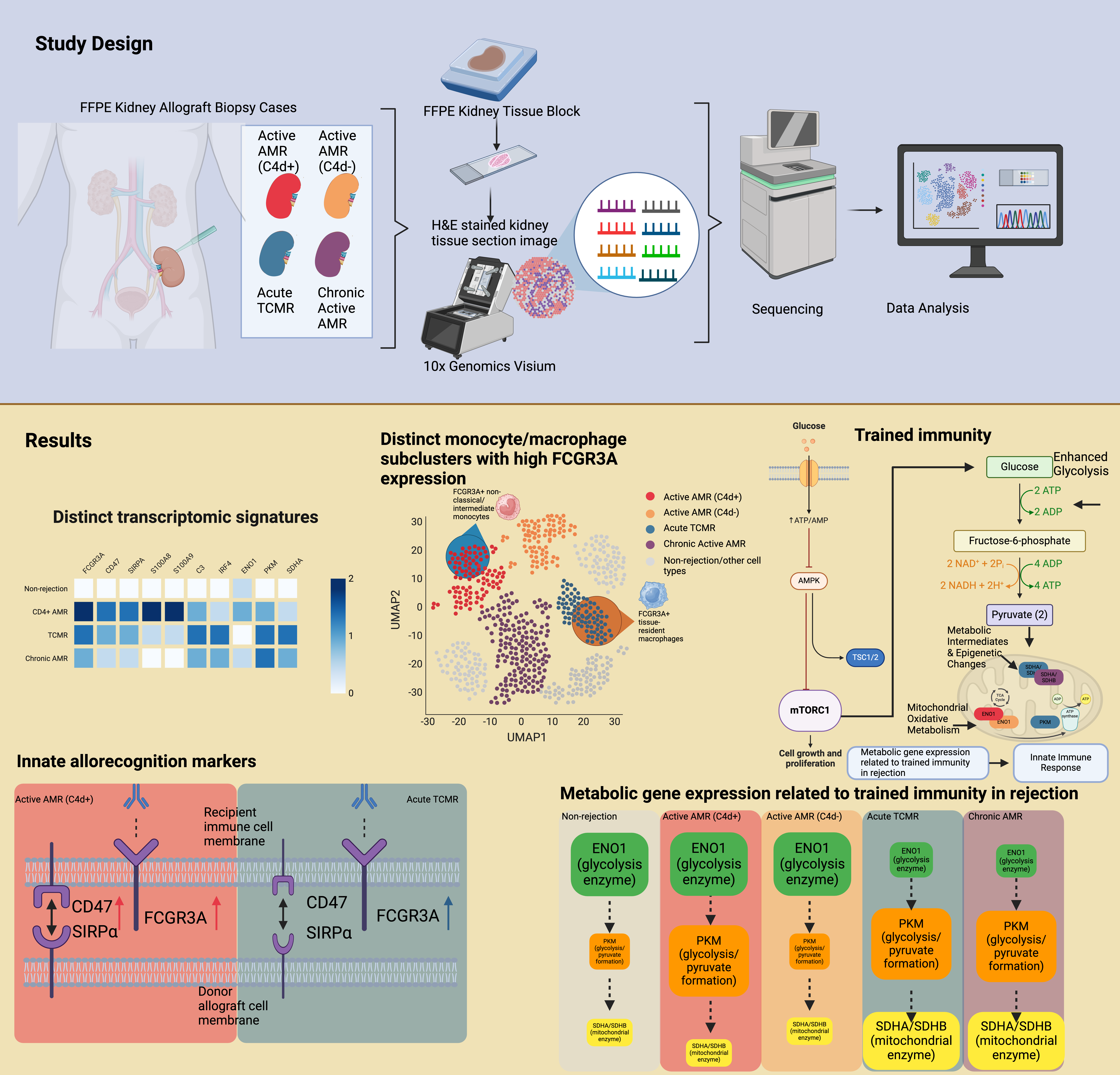

Supplement: Supplementary file 8 [file Image6.png]
